# The Influenza Vaccine May Protect Pregnant and Postpartum Women against Severe COVID-19

**DOI:** 10.3390/vaccines10020206

**Published:** 2022-01-28

**Authors:** Cristiane de Freitas Paganoti, Agatha Sacramento Rodrigues, Rossana Pulcineli Vieira Francisco, Rafaela Alkmin da Costa

**Affiliations:** 1 Division of Clinical Obstetrics, Hospital das Clinicas HCFMUSP, Faculty of Medicine, University of São Paulo, São Paulo 05403-000, Brazil; rafaela.alkmin@hc.fm.usp.br; 2 Department of Statistics, Federal University of Espírito Santo, Vitória 29075-910, Brazil; agatha.rodrigues@ufes.br; 3 Discipline of Obstetrics, Department of Obstetrics and Gynecology, Hospital das Clinicas HCFMUSP, Faculty of Medicine, University of São Paulo, São Paulo 05403-000, Brazil; rossana.francisco@hc.fm.usp.br

**Keywords:** pregnancy, COVID-19, influenza vaccines, maternal mortality

## Abstract

The SARS-CoV-2 pandemic has imposed a huge challenge on the antenatal care of pregnant women worldwide, with the maternal mortality rate being raised to alarming levels. While COVID-19 vaccines were developed, some studies highlighted a possible relationship between influenza vaccination and lower odds of COVID-19 infection. As obstetric patients belong to a high-risk group for respiratory diseases, this study evaluated whether influenza vaccination reduces the severity of COVID-19 infection and mortality among pregnant and postpartum women. We conducted a retrospective cohort study on 3370 pregnant and postpartum women from the Brazilian national database, where they were grouped according to their influenza vaccination status before the onset of COVID-19 symptoms. The intensive care unit admission and intubation rates were significantly higher among subjects in the unvaccinated group (*p* = 0.002 and *p* < 0.001, respectively). The odds of mortality risk among those who received the vaccine was 0.33, with a 95% confidence interval of 0.23–0.47. The numbers of patients who needed to be vaccinated to avoid a case of intensive care unit admission, intubation, or death due to COVID-19 were 11, 15, and 11, respectively. Influenza vaccines could confer protection against severe COVID-19 infection in pregnant and postpartum women.

## 1. Introduction

The severe acute respiratory syndrome coronavirus 2 (SARS-CoV-2) pandemic has imposed a huge challenge on the antenatal care of pregnant women worldwide, especially in Brazil [1]. Consequently, access to both health services and specialized care was hindered and the maternal mortality rate rose to alarming levels [2]. 

According to the Brazilian Obstetric Observatory (OOBr), 1948 Brazilian pregnant and postpartum women have succumbed to coronavirus disease 2019 (COVID-19) since the beginning of the pandemic. A total of 1488 maternal deaths were documented in 2021, which corresponded to a 223% increment from 2020. The percentage of severe cases also rose from 7.2% in 2020 to an alarming 14% in 2021 [3].

While the Brazilian COVID-19 vaccination program started in January 2021, pregnant and postpartum women were not included initially. Those aged 18 years or older who had comorbidities were only included from May 2021, and all aged 18 years or older were only included from July 2021 [4,5,6]. 

While COVID-19 vaccines were developed, some studies highlighted possible relationships between influenza vaccination and lower odds of COVID-19 infection, lower COVID-19 severity, and lower mortality rates [7,8,9,10,11,12,13,14,15,16,17,18,19]. However, other studies could not demonstrate such associations [20,21,22]. A possible conferment of cross-immunity by the influenza vaccination was speculated to stimulate an innate immune response that could offer some protection against COVID-19, known as trained innate immunity [19]. As physiological changes in the immune system during pregnancy pose a high risk for respiratory infections perinatally [23,24], pregnant and postpartum women might benefit from this cross-immunity boost provided by influenza vaccination.

Given that the influenza vaccine is safe throughout gestation and could be routinely administered at any stage of pregnancy [24,25,26,27], the aim of this study was to evaluate whether influenza vaccination reduces the severity and mortality of COVID-19 infection and mortality among pregnant and postpartum women.

## 2. Materials and Methods

This is a retrospective cohort study using the statistics from the Influenza Epidemiological Surveillance Information System, SIVEP-Gripe (Sistema de Informação de Vigilância Epidemiológica da Gripe) database. 

The SIVEP-Gripe is a nationwide surveillance database created in 2000 to monitor severe acute respiratory infections and collect data on virus circulation and respiratory infections in Brazil. Individuals presenting with fever, cough, and/or sore throat in sentinel monitoring units were documented in the database. In view of the H1N1 pandemic in 2009, a rigorous surveillance of cases of severe acute respiratory syndrome (SARS) was adopted, with compulsory notification of all SARS cases. SARS was defined as the presence of fever, cough, and dyspnea. From 2010, only hospitalized cases of SARS, both in public and private hospitals, as well as cases of deaths caused by SARS, irrespective of hospitalization, were to be reported. As virus surveillance for public health purposes has dynamic characteristics, frequent updates are made in the notification guidelines. At the time of the COVID-19 pandemic, cases of SARS with the presence of at least two of the following symptoms must be reported: fever, chills, sore throat, headache, cough, runny nose, olfactory or taste disorders, plus dyspnea or chest pressure, or saturation less than 95% (or blue coloration of the lips or face). Only cases of hospitalized SARS or SARS-related deaths must be reported.

The SIVEP-Gripe, a free and publicly accessible database (available at https://opendatasus.saude.gov.br/dataset/bd-srag-2020 and https://opendatasus.saude.gov.br/dataset/bd-srag-2021, accessed on 12 October 2021), records demographic, clinical, and epidemiological data, as well as laboratory/etiological results. There is also information on hospital admission and disease progression (recovery or death). 

This study aimed to evaluate the clinical and epidemiological outcomes and death rates of pregnant and postpartum women with severe SARS-CoV-2-induced SARS who did or did not receive influenza vaccination before the onset of COVID-19 symptoms. Data were obtained from Brazilian women enrolled in the epidemiological study between 16 February 2020 and 1 May 2021, i.e., week 8 of 2020 to week 17 of 2021.

Patients were selected according to the following inclusion criteria: pregnant and postpartum women of a childbearing age (10–55 years old), positive COVID-19 confirmed by reverse transcription–polymerase chain reaction (RT-PCR) SARS-CoV-2 or antigen, negative RT-PCR or antigen for influenza, completion of final outcome (recovery or death), and influenza vaccination before the onset of COVID-19 symptoms. Since this was a retrospective study, no exclusion criteria were determined.

Considering the retrospective nature of the study, as well as the anonymity of SIVEP-GRIPE, no prior informed consent or approval by the institutional ethics committee were necessary [28]. 

### Statistical Analyses 

Enrolled patients were grouped according to their influenza vaccination status, namely vaccinated and unvaccinated.

Categorical variables were presented as absolute and relative frequencies and were compared using the Chi-squared test or Fisher’s exact test, whichever was applicable. An odds ratio (OR) and a 95% confidence interval (CI 95%) were considered as measures of association. Continuous variables were presented as mean and standard deviation (SD) and were compared using the Student’s *t*-test. *p* < 0.005 was considered statistically significant. 

Propensity score matching (PSM) was used to estimate and assess balancing weights for the observations to balance the groups in relation to the confounding variables. The propensity score was estimated using logistic regression, and 1:1 matching was performed by considering the nearest neighbor matching [29]. The average treatment effect for the treated (ATT) was estimated for the vaccinated group effect, and the control variables considered were white ethnicity, age, education, and asthma. The results of PSM and standardized difference after matching are shown in the Supplemental Material.

The analyses were performed using the free statistical software R (R Foundation for Statistical Computing Platform, version 4.0.3 [30]), and PSM was carried out with the R MatchIt package [31].

Only valid responses for each variable were considered, and the percentage of missing data depended on the variable in question. The valid number of cases for each variable was stated in the tables.

The number needed to treat (NNT) was calculated, and the cost value for influenza vaccines [32] and the intensive care unit (ICU) daily rate [33] under the Brazilian health system were estimated.

## 3. Results

### 3.1. Study Population

**Figure 1:**
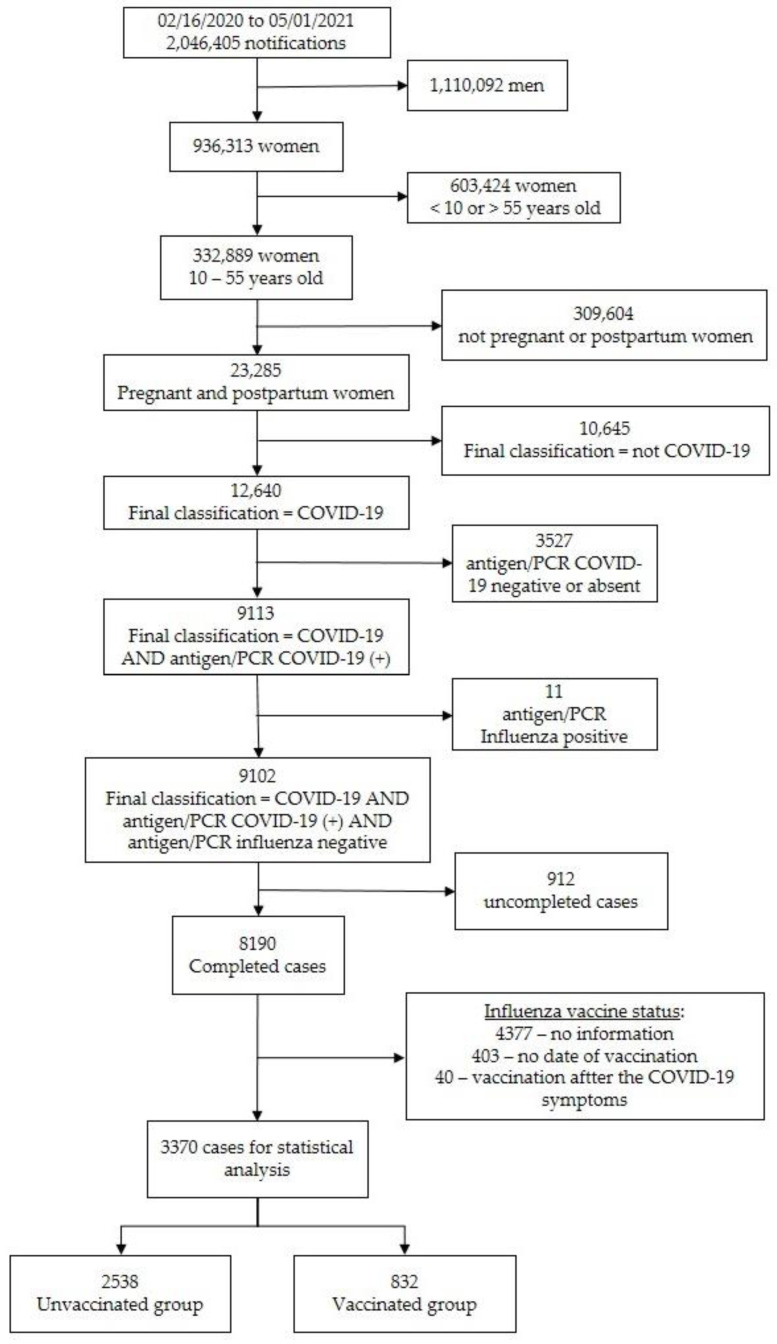
Flow chart depicting the enrollment process, explaining how the final number of subjects was derived for evaluation.

A total of 3370 patients were enrolled during the study period, according to the inclusion (Figure 1), and was divided into two groups according to their influenza vaccination status: vaccinated (*n* = 832; 24.7%) and non-vaccinated (*n* = 2538; 75.3%) groups.

### 3.2. Baseline Characteristics of the Subjects Enrolled

The baseline characteristics of the subjects are presented in Table 1. Women in the vaccinated group were younger than those in the unvaccinated group (*p* = 0.023), but the effect size was very small, as evidenced by the d-Cohen coefficient (d = 0.009 ([95% confidence interval {CI}: 0.01–0.017]). The non-whites were more unlikely to have received the influenza vaccine than the whites, by 1.32-fold (*p* = 0.001).

Asthma was the only disease presenting statistical difference between groups and was more frequent among women in the vaccinated group compared to those in the unvaccinated group (12.2% vs. 7.3%; *p* = 0.004), with an odds ratio (OR) of 1.77 (95% CI 1.20–2.59).

Considering the education levels of the subjects, there were fewer who studied up to 9 years in the vaccinated group than in the unvaccinated group (17.9% vs. 25%; *p* < 0.001) and more who studied over 12 years in the vaccinated group than in the unvaccinated group (24.1% vs. 17.7%; *p* < 0.001).

### 3.3. Clinical Manifestations of COVID-19

Table 2 presents the characteristics of COVID-19 symptoms according to vaccination status. Women in the vaccinated group had 0.81 lower odds of having any respiratory symptoms than those in the unvaccinated group (95% CI 0.67–0.97). Moreover, influenza vaccination conferred protection against dyspnea (OR 0.73; 95% CI 0.62–0.86; *p* < 0.001) and desaturation (OR 0.69; 95% CI 0.58–0.83; *p* < 0.001). The two most typical COVID-19 symptoms, loss of olfactory sense and loss of taste, were more frequently documented among women in the vaccinated group than those in the unvaccinated group (31.2% vs. 20.9%; *p* < 0.001 and 29.7% vs. 20.2%; *p* < 0.001, respectively).

### 3.4. COVID-19 Adverse Outcomes

Table 3 presents the characteristics of COVID-19 outcomes according to vaccination status. Intensive care unit (ICU) admission rates were significantly lower among women in the vaccinated group than those in the unvaccinated group (21.1% vs. 26.6%; *p*= 0.002). While the influenza vaccine provided this protective effect (OR 0.74; 95% CI 0.61–0.90), it did not provide a protective effect in terms of the length of stay in the ICU between the groups (*p* = 0.798).

The intubation rate for those who needed ventilatory support was lower in the vaccinated group than in the unvaccinated group (8.8% vs. 14.3%; *p*< 0.001). Receiving the influenza vaccine shortened the chance for invasive ventilatory support (OR 0.58; 95% CI 0.44–0.77).

Women who received the influenza vaccine had a significantly lower mortality rate than those who did not receive it (5.3% vs. 12.8%; *p* < 0.001). The odds of succumbing to COVID-19 among subjects who received the influenza vaccine was 0.38 (95% CI 0.27–0.53) in comparison to those who did not receive it.

Propensity score matching (PSM) was conducted to account for possible confounding factors (ethnicity, asthma, and education) in order to verify whether the effect on the final outcome could be attributed to influenza vaccination. Although there was a statistically significant difference in age between the groups, it was not included as a confounding factor because its statistical significance could be mainly attributed to the large sample size, as suggested by the very small size effect coefficient (Cohen’s d = 0.09 (95% CI 0.01–0.17).

As shown in Table 3, the three main outcomes (ICU admission, intubation, and death) remained statistically less frequent in the vaccinated group than in the unvaccinated group after balancing the covariates using PSM analysis. 

Hence, for those who did not receive the influenza vaccination, the chance of ICU admission, intubation, and mortality was 1.61 (95% CI 1.28–2.04), 1.88 (95% CI 1.39–2.56), and 3.03 (95% CI 2.13–4.35) times greater than in the vaccinated group, respectively.

The numbers needed to treat (NNT) for ICU admission, intubation, and death were 11, 15, and 11, respectively. Under the Brazilian health system, an influenza vaccine costs BRL 15.00 (USD 2.73) while the daily rate in the ICU is BRL 1600.00 (USD 290.90). Considering that the length of time in the ICU for the unvaccinated group was 12.15 days, the final cost would amount to BRL 19,440.00 (USD 3534.55). Therefore, spending BRL 165.00 (USD 30.00) on influenza vaccination could potentially save BRL 19,440.00 (USD 3534.55) for ICU admission.

## 4. Discussion

In this study, pregnant and postpartum women with severe COVID-19 who received influenza vaccination presented a 38% reduction in the odds of ICU admission, a 47% reduction in the odds of invasive ventilatory support (intubation), and a 67% reduction in the odds of succumbing to the disease compared to those who did not receive the influenza vaccine before the onset of COVID-19 symptoms.

These results show that influenza vaccination could have some protective effects on the severity of the clinical manifestations of COVID-19 in the obstetric population, thereby reducing the chance of succumbing to the disease. To the best of our knowledge, this is the first study to address the association between influenza vaccination and maternal mortality due to severe COVID-19 infection.

Considering the unequal and scant distribution of COVID-19 vaccines worldwide, and one of the objectives (health and well-being, and a reduction in maternal mortality) of the sustainable development goals [34], there is a need to look for alternative and more readily available care for the obstetric population during this pandemic, including the conferment of some degree of protection by the influenza vaccine. 

Prior studies suggested that the resultant immunity granted by influenza vaccination should enhance immunity against SARS-CoV-2, with a nonspecific protection cell-mediated response, so-called trained innate immunity, that can be stimulated by live attenuated vaccination [10,12,17,35,36]. 

It should not be overlooked that these immunological changes have been described in non-pregnant individuals and that pregnant women undergo adaptive physiological changes in the immune system that can interfere with their immune responses to viruses and vaccines [23].

Previous studies also speculated that the possibly healthier behavior of individuals who chose to be vaccinated, including the adoption of basic social etiquette, such as wearing a mask and maintaining social distancing, could have an association with the observed protective effect of flu vaccination [9,17,18,35]. In addition, diseases, such as asthma, are risk factors for flu, and individuals who have comorbidities are part of the priority group for influenza vaccination and tend to be more favorable toward accepting it. In line with previous studies [10,11,13,17,19], the present study documented asthma as the major comorbidity presenting statistical differences between the vaccinated and unvaccinated groups, with a higher proportion of asthmatic individuals in the vaccinated group. Beyond that, pregnant and postpartum women are more prone to adopting and following safety measures that could offer security to their offspring. 

Indeed, such an association could have some bias, especially regarding the social and epidemiological aspects of the subjects enrolled. The present study showed some evidence of social disparities between the two groups, such as the higher frequency of non-white ethnicity and lower educational levels in the unvaccinated group. Hence, individuals with easier access to vaccination programs could also be speculated to have benefited from easier and better access to health services, thereby avoiding worse outcomes, such as death. Previous studies [11,13,19] have also reported a higher proportion of Caucasians among vaccinated subjects. 

Both issues (ethnicity and education level) have a noteworthy social impact on antenatal care, especially with the consideration of the relatively low flu vaccination rates among pregnant and postpartum women. Even though the World Health Organization recommends vaccination during pregnancy, given the high risk for severe disease and death, along with the poor obstetric outcomes [37], the compliance to vaccinate is still low. Only 24.7% of the enrolled subjects in the current study received the influenza vaccination, similar to the study by Candelli et al. [12] (24.9%), despite the free and standardized flu vaccination for the obstetric population in all Brazilian states. However, some studies did report a higher compliant rate of influenza vaccination, ranging from 31.2% to 67% [9,10,13,18].

In terms of clinical manifestations of SARS-CoV-2 infection, the present study found that the more severe symptoms, such as dyspnea and desaturation, were more prevalent among subjects in the unvaccinated group, while de La Cruz Conti et al. [13] did not find such an association. Influenza vaccination conferred protection against, and reduced the odds of, severe clinical features of COVID-19 in the obstetric population. These findings should be considered when implementing public health policies for pregnant women, as they are susceptible to more severe manifestations of respiratory infections due to the physiological changes during pregnancy. 

However, the two most typical symptoms of SARS-CoV-2 infection, loss of olfactory sense and loss of taste, appeared to be more common among the vaccinated subjects in the present study. While the immunological mechanisms remain unclear, it is speculated to be caused by infection of the epithelial cells in the olfactory bulb, which decreases the interferon response in the epithelium of the upper airway, mainly interferon alpha, beta, and lambda [38].

PSM was performed to minimize bias associated with observational and retrospective studies, so as to evaluate whether the protective effect in the final outcome could be assigned only to influenza vaccination. The results suggest that flu vaccination seems to have a protective effect on ICU admission rates, the need for invasive ventilator support (intubation), and mortality in the obstetric population. Vaccinated subjects with severe COVID-19 presented with a 37% reduction in ICU admission, a 47% reduction in the need for intubation, and a 67% reduction in death rate. In addition, there was a strong correlation between non-vaccination and death rates.

The NNT evaluation showed how enhancing the influenza vaccination coverage for the obstetric population can be cost-effective. From a public health perspective, implementing influenza vaccination could facilitate an optimization of health resources, as there was a reduction in ICU admissions, a treatment modality that is not always available and one that comes with a higher cost. Given that only 11 pregnant women have to be vaccinated in order to avoid one ICU admission, spending BRL 165.00 (USD 30.00) for influenza vaccination can potentially save BRL 19,440.00 (USD 3534,55) for ICU admission.

However, some study limitations need to be addressed. This is an observational retrospective cohort study based on a standard case report form, which means that it depends on an adequate and complete fulfillment of the form to gather all information. Consequently, subjects who did not disclose their vaccination status (53.4%) and those who received their vaccination after the onset of COVID-19 symptoms (0.4%) were excluded from the analysis. Additionally, 4.9% of those who completed the report did not state the date of their influenza vaccination and were, hence, also excluded from the analysis. Moreover, despite our efforts to mitigate clinical and socioeconomic biases that could interfere with the main results (propensity score matching was conducted to account for these possible confounding factors), some data, such as the income or the status for other live attenuated vaccines, were not available on the Brazilian SIVEP-Gripe form and, thus, we could not include them in the analysis.

Only those who received the vaccination before the onset of COVID-19 symptoms were included in the evaluation in order to guarantee that the vaccine had taken effect prior to the disease. Moreover, the subjects analyzed were rigorously selected with all completed data, mainly the etiologic confirmation of acute COVID-19 infection, the exclusion of H1N1 co-infection, and the status and date of flu vaccination. Key pieces of information, including differences in socioeconomic and healthcare features in all regions of the Brazilian states, were also considered.

Furthermore, only subjects with severe manifestations of COVID-19 were analyzed in the present study, since they make up the majority who could succumb to the disease and, hence, might benefit the most from measures undertaken to avoid it. As such, it is not known if influenza vaccination can benefit those with mild COVID-19 symptoms and prevent them from developing severe COVID-19. This was, however, not within the scope of this study, as the Brazilian database SIVEP-Gripe only gathers information for severe acute respiratory infections.

Our study suggests that influenza vaccination confers protection against COVID-19 severity in the obstetric population. Nonetheless, due to the retrospective and observational nature of the study, causability cannot be affirmed and the findings should be evaluated in more properly designed studies. Nonetheless, considering the severity of influenza infection in pregnant women, and given the safety and efficacy of the influenza vaccine in pregnant women, ethical issues may arise from randomized clinical trials and, therefore, only observational studies may be suitable to provide relevant information about this subject.

This pilot study could influence the conduct of more studies to verify the observed protective effect of influenza vaccination for pregnant and postpartum women and encourage the implementation of public health policies to facilitate higher compliance of flu vaccination during pregnancy. Furthermore, the COVID-19 pandemic is far from over with novel variants emerging, and there are seasonal burdens of influenza infection worldwide. Considering that non-elderly and non-pregnant populations, who are usually not eligible as priority groups for influenza vaccination, are frequently not included in the currently published studies [7,8,9,10,11,12,13,14,15,16,17,18,19], much like the possible association between influenza vaccination and less severe COVID-19 infection, studies on this subject could also help to enhance and stimulate the vaccination coverage of this population.

## 5. Conclusions

Administration of the influenza vaccine, before the onset of COVID-19 symptoms, could offer protective effects in pregnant and postpartum women presenting with severe COVID-19, thereby reducing ICU admission, intubation, and death.

## Figures and Tables

**Table 1:** Baseline characteristics of the subjects according to vaccination status.

Variables	Vaccinated (*n* = 832)	Unvaccinated (*n* = 2538)	*p*-Value
Age (years); median ± sd ^$^	29.81 ± 6.78	30.44 ± 7.27	*0.0234* ^1^
Ethnicity, *n* (%) White Non-white			*0.001* ^2^
	387/773 (50.1)	997/2308 (43.2)	
	386/773 (49.9)	1311/2308 (56.8)	
Comorbidities, *n* (%):			
Cardiac	61/371 (16.4)	149/1143 (13.0)	0.1181 ^2^
Diabetes *mellitus*	59/371 (15.9)	163/1158 (14.1)	0.4327 ^2^
Hematologic	6/359 (1.7)	14/1122 (1.2)	0.599 ^3^
Obesity	48/366 (13.1)	130/1145 (11.4)	0.4141 ^2^
Asthma	45/368 (12.2)	83/1135 (7.3)	*0.0047 ^2^*
Hepatic	1/360 (0.3)	11/1112 (1.0)	0.3134 ^3^
Neurologic	10/362 (2.8)	19/1121 (1.7)	0.2905 ^2^
Other lung diseases	9/362 (2.5)	21/1124 (1.9)	0.6086 ^2^
Immunosuppression	6/360 (1.7)	37/1120 (3.3)	0.1532 ^2^
Renal	5/358 (1.4)	13/1113 (1.2)	0.7825 ^3^
Education, *n* (%) Up to 9 years From 9 to 12 years Over 12 years			*<0.001* ^2^
	103/577 (17.9)	351/1406 (25.0)	
	335/577 (58.1)	806/1406 (57.3)	
	139/577 (24.1)	249/1406 (17.7)	
Residence area, *n* (%) Urban Periurban Rural			0.9147 ^3^
	749/790 (94.8)	2270/2388 (95.1)	
	2/790 (0.3)	8/2388 (0.3)	
	39/790 (4.9)	110/2388 (4.6)	

*n*: number; sd: standard deviation; ^1^, Student’s *t*-test; ^2^, Chi-square test; ^3^, Fisher’s exact test; ^$^, Cohen’s d = 0.09 (95% CI 0.01–0.17: very small).

**Table 2:** Characteristics of COVID-19 symptoms according to vaccination status.

Variables	Vaccinated (*n* = 832)	Unvaccinated (*n* = 2538)	OR (95% CI)
Symptoms, *n* (%)			
Fever	509/744 (66.6)	1529/2371 (64.5)	1.10 (0.93–1.31)
Cough	593/782 (75.8)	1808/2402 (75.3)	1.03 (0.85–1.24)
Sore Throat	233/727 (32.0)	664/2188 (30.3)	1.08 (0.90–1.30)
Dyspnea	418/759 (55.1)	1477/2358 (62.6)	***0.73 (0.62–0.86)***
Respiratory discomfort	411/748 (54.9)	1198/2269 (52.8)	1.09 (0.92–1.29)
Desaturation	243/734 (33.1)	938/2254 (41.6)	***0.69 (0.58–0.83)***
Diarrhea	121/716 (16.9)	329/2130 (15.4)	1.11 (0.89–1.40)
Vomit	109/718 (15.2)	303/2136 (14.2)	1.08 (0.85–1.37)
Abdominal pain	56/440 (12.7)	187/1597 (11.7)	1.10 (0.80–1.51)
Fatigue	160/450 (35.6)	516/1633 (31.6)	1.19 (0.96–1.49)
Loss of olfactory sense	145/465 (31.2)	340/1628 (20.9)	***1.72 (1.36–2.16)***
Loss of taste	137/461 (29.7)	329/1631 (20.2)	***1.67 (1.32–2.11)***
Any respiratory symptom	570/783 (72.8)	1865/2427 (76.8)	***0.81 (0.67–0.97)***
Any symptom	781/816 (95.7)	2415/2517 (95.9)	0.94 (0.64–1.40)

CI: confidence interval; *n*: number; OR: odds ratio. Bold and Italic–statistical significance

**Table 3:** Characteristics of COVID-19 outcomes according to vaccination status—overall and after propensity score matching.

Variables *n*(%)	Vaccinated (*n* = 832)	Unvaccinated (*n* = 2.538)	OR (95% CI)	Vaccinated (*n* = 832) PSM	Unvaccinated (*n* = 832) PSM	OR (95% CI) PSM
ICU admission	160/757 (21.1)	629/2362 (26.6)	***0.74*** ***(0.61–0.90)***	160/757 (21.1)	237/782 (30.3)	***0.62 (0.49–0.78)***
Intubation	68/769 (8.8)	336/2355 (14.3)	***0.58*** ***(0.44–0.77)***	68/769 (8.8)	122/786 (15.5)	***0.53 (0.39–0.72)***
Final outcome, Cure Death	788/832 (94.7) 44/832 (5.3)	2213/2538 (87.2) 321/2538 (12.8)	***0.38*** ***(0.27–0.53)***	788/832 (94.7) 44/832 (5.3)	711/832 (85.5) 121/832 (14.5)	***0.33 (0.23–0.47)***

CI: confidence interval; *n*: number; OR: odds ratio; PSM: propensity score matching. Bold and Italic–statistical significance

## Data Availability

The data and R codes that were used to support the findings of this study are available in the GitHub repository at https://github.com/observatorioobstetrico/Paper_Influenza_Vaccine. These data were derived in August 2021 from the following resources available in the public domain: https://opendatasus.saude.gov.br/dataset/bd-srag-2020 and https://opendatasus.saude.gov.br/dataset/bd-srag-2021 (accessed on 1 October 2021).

## Supplementary Materials

The following supporting information can be downloaded at: https://www.mdpi.com/article/10.3390/vaccines10020206/s1, Text S1: Documentation of the article ‘Influenza vaccine confers protection against severe COVID-19 perinatally’.
