# Supplementary material for: The Influenza Vaccine May Protect Pregnant and Postpartum Women against Severe COVID-19
Source: Vaccines (Basel). 2022 Jan 28;10(2):206. doi: 10.3390/vaccines10020206 (PMC8875780; doi:10.3390/vaccines10020206)
Supplement: Supplementary file 1 [file vaccines-10-00206-s001.zip › vaccines-1536060-supplementary.pdf]

Documentation of the article 'Influenza vaccine confers protection against severe COVID-19 perinatally'

Codes and outputs

December 02, 2021

Sumário

Description 2

About the database and R packages used 2

Case selection and data treatment 4

Epidemiologic characteristics 10

Comorbities 15

Symptoms 24

Outcome 34

Time between covid vaccine and first symptoms of covid-19 38

Propensity Scoring Matching (PSM) 39

Vaccination and mortality maps 65

Description

This file presents the documentation of the analysis of article "Influenza vaccine confers protection against severe COVID-19 perinatally".

About the database and R packages used

The data are analyzed using the free-software R (https://www.R-project.org) in version 4.0.3. Next, we present and load the libraries used in the data analysis process.

```
#load packages
loadlibrary <- function(x) {
  if (!require(x, character.only = TRUE)) {
    install.packages(x, dependencies = T)
    if (!require(x, character.only = TRUE))
      stop("Package not found")
  }
}

packages <-
  c(
    "readr",
    "readxl",
    "janitor",
    "dplyr",
    "forcats",
    "stringr",
    "lubridate",
    "summarytools",
    "magrittr",
    "questionr",
    "knitr",
    "data.table",
    "modelsummary",
    "kableExtra",
    "DescTools",
    "ggplot2",
    "effectsize",
    "WeightIt",
    "MatchIt",
    "ggplot2",
    "ggpubr"
  )

lapply(packages, loadlibrary)
```

One can see below the functions that will be used in the data analysis.

```
#functions for summary measures
media <- function(x)
  mean(x, na.rm = TRUE)
mediana <- function(x)
```

```
  median(x, na.rm = TRUE)
DP <- function(x)
  sd(x, na.rm = TRUE)
minimo <- function(x)
  base::min(x, na.rm = TRUE)
maximo <- function(x)
  base::max(x, na.rm = TRUE)
q25 <- function(x)
  stats::quantile(x, p = 0.25, na.rm = TRUE)
q75 <- function(x)
  stats::quantile(x, p = 0.75, na.rm = TRUE)
IQR <- function(x)
  round(q75(x) - q25(x), 2)
n <- function(x)
  sum(!is.na(x))
```

This is a retrospective cohort study using the data from the Influenza Epidemiological Surveillance Information System, SIVEP-Gripe (Sistema de Informação de Vigilância Epidemiológica da Gripe) database.

The SIVEP-Gripe is a nationwide surveillance database created to monitor severe acute respiratory infections and data on virus circulation and respiratory infections in Brazil. Flu cases should be notified for individuals presenting fever and cough and/or sore throat in sentinel monitoring units. In 2009, with the burden of the H1N1 pandemic, a rigorous surveillance of the cases of severe acute respiratory syndrome (SARS) was adopted, with compulsory notification of all SARS cases. The definition of SARS included the presence of fever, cough and dyspnea. Since 2010, only hospitalized cases of SARS must be notified, both in public and in private hospitals, as well as cases of deaths caused by SARS, irrespective of hospitalization. Once virus surveillance for public health purposes has dynamic characteristics, frequent updates are made in notification guidelines. At the time of COVID-19 pandemic, cases of SARS must be notified in the presence of at least two of the following symptoms: fever, chills, sore throat, headache, cough, runny nose, olfactory or taste disorders PLUS dyspnea or chest pressure or saturation less than 95% or blue coloration of lips or face. Only cases of hospitalized SARS or SARS-related death must be notified. The SIVEP-Gripe records contain demographic, clinical and epidemiological data, as well as laboratory/etiological results. There is also information about hospital admission and disease progression (recovery or death).

The period analyzed comprises epidemiological data from 2020, with a database obtained on August 10, 2021 on the website https://opendatasus.saude.gov.br/dataset/bd-srag-2020, and from 2021, with a database obtained on August 10, 2021 on the website https://opendatasus.saude.gov.br/dataset/bd-srag-2021. These datasets can be obtained at https://drive.google.com/drive/folders/1s3SwBWlCRS9pkEsv2sWftA-qGqJ_jTA1?usp=sharing. They are loaded and combined below:

```
#loading the datasets
#2021
dados_a <- read_delim(
  "INFLUD21-02-08-2021.csv",
  ";",
  escape_double = FALSE,
  locale = locale(encoding = "ISO-8859-2"),
  trim_ws = TRUE
)

#2020
dados_b <- read_delim(
  "INFLUD-02-08-2021.csv",
  ";",
```

```
  escape_double = FALSE,
  locale = locale(encoding = "ISO-8859-2"),
  trim_ws = TRUE
)


#### Concatenating 2020 and 2021 datasets ##############
dados_a <- dados_a %>%
  mutate(FATOR_RISC = case_when(FATOR_RISC == 1 ~ "S",
                                FATOR_RISC == 2 ~ "N"))
dados_b <- dados_b %>%
  mutate(FATOR_RISC = case_when(FATOR_RISC == 1 ~ "S",
                                FATOR_RISC == 2 ~ "N"))

# dados_b$OBES_IMC <- as.numeric(dados_b$OBES_IMC)

dados <- full_join(dados_a, dados_b)

#Create case year variable
dados <-  dados %>%
  dplyr::mutate(
    dt_sint = as.Date(DT_SIN_PRI, format = "%d/%m/%Y"), #date of first symptoms
    dt_nasc = as.Date(DT_NASC, format = "%d/%m/%Y"), #date of birth
    dt_vac_gripe = as.Date(DT_UT_DOSE, format = "%d/%m/%Y"), #date of Influenza vaccine
    ano = lubridate::year(dt_sint), #year of the case
  )
```

There are 2528374 observations in the database. To see the dictionary of variables, access (in Portuguese): https://opendatasus.saude.gov.br/dataset/ae90fa8f-3e94-467e-a33f-94adbb66edf8/resource/8f571374-c555-4ec0-8e44-00b1e8b11c25/download/dicionario-de-dados-srag-hospitalizado-27.07.2020-final.pdf

Case selection and data treatment

The first filter is to select cases from February 16, 2020 (8th epidemiological week of symptoms of 2020) to May 1, 2021 (epidemiological week 17 of 2021).

```
#selection of cases from February 16, 2020 (8th epidemiological week of symptoms of 2020)
#to May 1, 2021 (week 17 of 2021).
sem <- 17

dados1 <- dados %>%
  filter((ano == 2020 & SEM_PRI >=8) | ano == 2021)

#week 53 has the first two days of 2021 and the year is 2021, but it's actually 2020.
dados1 <- dados1 %>%
  mutate(ano = ifelse(ano == 2021 & SEM_PRI ==53, 2020, ano)) %>%
  filter(ano == 2020 | (ano == 2021 & SEM_PRI <= sem))
```

There are 2046405 observations in the database after selection of valid years.

The next selection is female:

```
#filtering F
dados2 <- filter(dados1, CS_SEXO == "F")
```

There are 936313 observations in the database.

Selection of women of childbearing age (10 to 55 years):

```
#creating the age variable as the difference between dt_sint and dt_nasc.
#In cases without dt_nasc, we consider
#the NU_AGE_N field
dados2 <- dados2 %>%
  mutate(
         idade = as.period(interval(start = dt_nasc, end = dt_sint))$year,
         age = ifelse(is.na(idade), NU_IDADE_N, idade)
  )

#Filtering of cases aged 55 and under
dados3 <- dados2 %>%
  filter(age > 9 & age <= 55)
```

There are 332889 observations in the database.

The next step is to identify pregnant and postpartum people (variable `classi_gesta_puerp`) and then select only those cases.

```
#Creating the classification variable if pregnant, postpartum and
##neither pregnant nor postpartum
dados3 <- dados3 %>%
  mutate(
    classi_gesta_puerp = case_when(
      CS_GESTANT == 1  ~ "1tri",
      CS_GESTANT == 2  ~ "2tri",
      CS_GESTANT == 3  ~ "3tri",
      CS_GESTANT == 4  ~ "IG_ig",
      CS_GESTANT == 5 &
        PUERPERA == 1 ~ "puerp",
      CS_GESTANT == 9 & PUERPERA == 1 ~ "puerp",
      TRUE ~ "não" #no
    )
  )

freq(dados3$classi_gesta_puerp)
```

```
##              n    % val%
## 1tri      2034  0.6  0.6
## 2tri      4815  1.4  1.4
## 3tri     10477  3.1  3.1
## IG_ig      964  0.3  0.3
## não     309604 93.0 93.0
## puerp     4995  1.5  1.5
```

```
#filtering only pregnant and postpartum women
dados4 <- dados3 %>%
  filter(classi_gesta_puerp != "não")
```

There are 23285 observations in the database.

We selected only confirmed cases of COVID-19.

```
dados4 <- dados4 %>%
  mutate(
    classi_fin = case_when(
      CLASSI_FIN == 5 ~ "covid",
      TRUE ~ "não" #no
    )
  )


#filtering only covid cases
dados5 <- dados4 %>%
    filter(CLASSI_FIN == 5)
```

There are 12640 observations in the database.

Now let's select the cases of COVID by PCR or antigen, but which are also not positive for Influenza.

```
#COVID case diagnosed by PCR
dados5 <- dados5 %>%
  mutate(pcr_covid_SN = case_when(
    (PCR_SARS2 == 1) |
      (
        str_detect(DS_PCR_OUT, "SARS|COVID|COV|CORONA|CIVID")
      ) ~ "sim", #yes
    TRUE ~ "não" #no
  ))

#Influenza case diagnosed by PCR
dados5 <- dados5 %>%
  mutate(pcr_influenza_SN = case_when(
    (POS_PCRFLU == 1) |
      (
        str_detect(DS_PCR_OUT, "INFLU|INFLUENZA")
      ) ~ "sim", #yes
    TRUE ~ "não" #no
  ))

with(dados5, table(pcr_influenza_SN,pcr_covid_SN))
```

```
##                   pcr_covid_SN
## pcr_influenza_SN  não  sim
##               não 4603 8027
##               sim    0   10
```

There are 10 cases that are positive for COVID and for Influenza by PCR.

```
#Case of COVID diagnosed by antigen
dados5 <- dados5 %>%
  mutate(antigenio_covid_SN = case_when(
    (AN_SARS2 == 1) |
```

```
      (
        str_detect(DS_AN_OUT, "SARS|COVID|COV|CORONA|CIVID")
      ) ~ "sim", #yes
    TRUE ~ "não" #no
  ))


#Influenza case diagnosed by antigen
dados5 <- dados5 %>%
  mutate(antigenio_influenza_SN = case_when(
    (POS_AN_FLU == 1) |
      (
        str_detect(DS_AN_OUT, "INFLU|INFLUENZA")
      ) ~ "sim", #yes
    TRUE ~ "não" #no
  ))

with(dados5, table(antigenio_influenza_SN, antigenio_covid_SN))
```

```
##                       antigenio_covid_SN
## antigenio_influenza_SN   não   sim
##                    não 11413  1226
##                    sim     1     0
```

There are no positive cases for COVID and for Influenza by antigen.

We will now select the cases of COVID confirmed by PCR or antigen.

```
with(dados5, table(pcr_covid_SN, antigenio_covid_SN))
```

```
##             antigenio_covid_SN
## pcr_covid_SN  não  sim
##          não 3527 1076
##          sim 7887  150
```

```
#filtering only covid cases by PCR or antigen
dados6 <- dados5 %>%
    filter(pcr_covid_SN == "sim" | antigenio_covid_SN == "sim")
```

There are 9113 observations in the database.

Now it's time to remove cases that are also positive for Influenza.

```
with(dados6, table(pcr_influenza_SN, antigenio_influenza_SN))
```

```
##                 antigenio_influenza_SN
## pcr_influenza_SN  não  sim
##              não 9102    1
##              sim   10    0
```

```r
#filtering only negative cases of Influenza by PCR or antigen
dados7 <- dados6 %>%
    filter(pcr_influenza_SN != "sim" & antigenio_influenza_SN != "sim") #'sim' means 'yes'
```

There are 9102 observations in the database.

Finally, we will only select the finalized cases (death or cure). The variable that indicates the outcome is `EVOLUCAO`, with the categories: 1-Cure; 2-Death; 3- Death from other causes; 9-Ignored.

```r
with(dados7, freq(EVOLUCAO))
```

```
##       n    % val%
## 1  7185 78.9 84.6
## 2   991 10.9 11.7
## 3    14  0.2  0.2
## 9   302  3.3  3.6
## NA  610  6.7   NA
```

Let's select only the finalized cases:

```r
#filtering only completed cases
dados8 <- dados7 %>%
  filter((EVOLUCAO == 1 | EVOLUCAO == 2 | EVOLUCAO == 3) & !is.na(EVOLUCAO))


#creating the evolution variable
dados8 <- dados8 %>%
  mutate(death = case_when(
    EVOLUCAO == 1 ~ "cure",
    EVOLUCAO == 2 ~ "death",
    EVOLUCAO == 3 ~ "death"
  ))

with(dados8, freq(death))
```

```
##          n    % val%
## cure  7185 87.7 87.7
## death 1005 12.3 12.3
```

There are 8190 observations in the database.

Vaccination variable analysis

Let's look at the variable that indicates whether the person was vaccinated for H1n1 (variable `vaccine`).

```r
#recoding the influenza vaccine variable
dados8 <- dados8 %>%
  mutate(vaccine = case_when(
    VACINA == 1 ~ "yes",
    VACINA == 2 ~ "no",
    TRUE ~ NA_character_
```

```
  ))
#Influenza vaccine indicator frequency table
with(dados8, freq(vaccine))
```

```
##         n    % val%
## no  2538 31.0 66.6
## yes 1275 15.6 33.4
## NA  4377 53.4   NA
```

Let's now create the time variable between the Influenza vaccine (for those who took it) and the first symptoms of COVID-19.

```
dados8 <- dados8 %>%
  mutate(
    time_vaccine_symptoms = as.numeric(dt_sint - dt_vac_gripe)
  )

dados8_aux <- dados8 %>%
  filter(vaccine == "no")
sum(is.na(dados8_aux$time_vaccine_symptoms))
```

```
## [1] 2538
```

```
dados8_aux <- dados8 %>%
  filter(is.na(vaccine))
sum(is.na(dados8_aux$time_vaccine_symptoms))
```

```
## [1] 4377
```

```
dados8_aux <- dados8 %>%
  filter(vaccine == "yes")
summary(dados8_aux$time_vaccine_symptoms)
```

```
##    Min. 1st Qu.  Median    Mean 3rd Qu.    Max.    NA's
##   -41.0    27.0    79.5   113.0   177.0   687.0     403
```

Note that when `vaccine = no` there is no information about time between vaccine and symptoms, as expected.

Among those vaccinated, there are 403 pregnant and postpartum women without information on the date of vaccination. Note that there is still negative data (date of first symptom was less than date of Influenza vaccination).

The next step is filtering cases that the vaccine was applied before the first symptoms of COVID-19 for those who have been vaccinated.

```
#Filtering positive time_vaccine_symptoms (vaccine before sympthoms)
data_final <- dados8 %>%
  filter(vaccine == "no" | (vaccine == "yes" & time_vaccine_symptoms > 0))
```

```
with(data_final, freq(vaccine))
```

```
##          n     % val%
## no    2538 75.3 75.3
## yes    832 24.7 24.7
```

Epidemiologic characteristics

```
# Ethnicity
data_final <-  data_final %>%
  mutate(
    ethnicity = case_when(
      CS_RACA == 1 ~ "white",
      CS_RACA == 2 ~ "black",
      CS_RACA == 3 ~ "yellow",
      CS_RACA == 4 ~ "brown",
      CS_RACA == 5 ~ "indigenous",
      TRUE ~ NA_character_
    ),
    white_color = case_when(
      ethnicity == "white" ~ "yes",
      is.na(ethnicity) ~ NA_character_,
      TRUE ~ "no"
    )
  )

# Education
data_final <-  data_final %>%
  mutate(
    education = case_when(
      CS_ESCOL_N == 0 ~ "no school",
      CS_ESCOL_N == 1 ~ "fund1",
      CS_ESCOL_N == 2 ~ "fund2",
      CS_ESCOL_N == 3 ~ "high school",
      CS_ESCOL_N == 4 ~ "college",
      TRUE ~ NA_character_
    ),
    education2 = case_when(
      CS_ESCOL_N <= 2 ~ "up to 9 years",
      CS_ESCOL_N == 3 ~ "from 9 to 12 years",
      CS_ESCOL_N == 4 ~ "over 12 years",
      TRUE ~ NA_character_
    )
  )
data_final$education2 <-
  factor(data_final$education2, levels = c("up to 9 years", "from 9 to 12 years", "over 12 years"))

# Age group
data_final <-  data_final %>%
  mutate(
```

```
    age_group = case_when(
      age <= 19 ~ "<20",
      age >= 20
      & age <= 34 ~ "20-34",
      age >= 35 ~ ">=35",
      TRUE ~ NA_character_
    )
  )
data_final$age_group <-
  factor(data_final$age_group, levels = c("<20", "20-34", ">=35"))


# residence area
data_final <-  data_final %>%
  mutate(
    residence = case_when(
      CS_ZONA == 1 ~ "urban",
      CS_ZONA == 2 ~ "rural",
      CS_ZONA == 3 ~ "periurban",
      TRUE ~ NA_character_
    )
  )
```

Ethnicity

```
with(data_final, ctable(ethnicity, vaccine,  prop = "c", useNA = "no", chisq = FALSE, OR = TRUE))
```

```
## Cross-Tabulation, Column Proportions
## ethnicity * vaccine
## Data Frame: data_final
##
## ------------ --------- --------------- -------------- ---------------
##                vaccine              no            yes           Total
##    ethnicity
##        black               134 (  5.8%)    49 (  6.3%)    183 (  5.9%)
##        brown              1141 ( 49.4%)   325 ( 42.0%)   1466 ( 47.6%)
##   indigenous                 9 (  0.4%)     5 (  0.6%)     14 (  0.5%)
##        white               997 ( 43.2%)   387 ( 50.1%)   1384 ( 44.9%)
##       yellow                27 (  1.2%)     7 (  0.9%)     34 (  1.1%)
##        Total              2308 (100.0%)   773 (100.0%)   3081 (100.0%)
## ------------ --------- --------------- -------------- ---------------
```

```
fisher.test(data_final$ethnicity, data_final$vaccine)
```

```
##
##  Fisher's Exact Test for Count Data
##
## data:  data_final$ethnicity and data_final$vaccine
## p-value = 0.005438
## alternative hypothesis: two.sided
```

White color

```r
with(data_final, ctable(white_color, vaccine,  prop = "c", useNA = "no", chisq = TRUE, OR = TRUE))
```

```
## Cross-Tabulation, Column Proportions
## white_color * vaccine
## Data Frame: data_final
##
##
## ------------- --------- --------------- -------------- ---------------
##                 vaccine              no            yes           Total
##   white_color
##            no               1311 ( 56.8%)   386 ( 49.9%)   1697 ( 55.1%)
##           yes                997 ( 43.2%)   387 ( 50.1%)   1384 ( 44.9%)
##         Total               2308 (100.0%)   773 (100.0%)   3081 (100.0%)
## ------------- --------- --------------- -------------- ---------------
##
## ----------------------------
##  Chi.squared   df   p.value
## ------------- ---- ---------
##    10.7608     1     0.001
## ----------------------------
##
## ----------------------------------
##  Odds Ratio   Lo - 95%   Hi - 95%
## ------------ ---------- ----------
##     1.32        1.12       1.55
## ----------------------------------
```

Education

```r
with(data_final, ctable(education, vaccine, prop = "c", useNA = "no", chisq = FALSE, OR = TRUE))
```

```
## Cross-Tabulation, Column Proportions
## education * vaccine
## Data Frame: data_final
##
## ------------- --------- --------------- -------------- ---------------
##                 vaccine              no            yes           Total
##     education
##       college                249 ( 17.7%)   139 ( 24.1%)    388 ( 19.6%)
##         fund1                106 (  7.5%)    25 (  4.3%)    131 (  6.6%)
##         fund2                237 ( 16.9%)    77 ( 13.3%)    314 ( 15.8%)
##   high school                806 ( 57.3%)   335 ( 58.1%)   1141 ( 57.5%)
##     no school                  8 (  0.6%)     1 (  0.2%)      9 (  0.5%)
##         Total               1406 (100.0%)   577 (100.0%)   1983 (100.0%)
## ------------- --------- --------------- -------------- ---------------
```

Education (years)

```
with(data_final, ctable(education2, vaccine, prop = "c", useNA = "no", chisq = TRUE, OR = TRUE))
```

```
## Cross-Tabulation, Column Proportions
## education2 * vaccine
## Data Frame: data_final
##
##
## -------------------- --------- --------------- -------------- ---------------
##                        vaccine              no            yes           Total
##           education2
##         up to 9 years                351 ( 25.0%)   103 ( 17.9%)    454 ( 22.9%)
##    from 9 to 12 years                806 ( 57.3%)   335 ( 58.1%)   1141 ( 57.5%)
##        over 12 years                 249 ( 17.7%)   139 ( 24.1%)    388 ( 19.6%)
##                Total                1406 (100.0%)   577 (100.0%)   1983 (100.0%)
## -------------------- --------- --------------- -------------- ---------------
##
## ----------------------------
##  Chi.squared   df   p.value
## ------------- ---- ---------
##    17.592      2     2e-04
## ----------------------------
```

Gestational moment

```
with(data_final, ctable(classi_gesta_puerp, vaccine, prop = "c", useNA = "no", chisq = TRUE, OR = TRUE)
```

```
## Cross-Tabulation, Column Proportions
## classi_gesta_puerp * vaccine
## Data Frame: data_final
##
##
## -------------------- --------- --------------- -------------- ---------------
##                        vaccine              no            yes           Total
##   classi_gesta_puerp
##                 1tri             213 (  8.4%)    49 (  5.9%)    262 (  7.8%)
##                 2tri             587 ( 23.1%)   173 ( 20.8%)    760 ( 22.6%)
##                 3tri            1181 ( 46.5%)   457 ( 54.9%)   1638 ( 48.6%)
##               IG_ig               77 (  3.0%)    14 (  1.7%)     91 (  2.7%)
##               puerp              480 ( 18.9%)   139 ( 16.7%)    619 ( 18.4%)
##               Total             2538 (100.0%)   832 (100.0%)   3370 (100.0%)
## -------------------- --------- --------------- -------------- ---------------
##
## ----------------------------
##  Chi.squared   df   p.value
## ------------- ---- ---------
##    21.5466     4     2e-04
## ----------------------------
```

Age

```
datasummary((vaccine) ~ age*(n+media+DP+mediana+q25+q75+IQR),
            data = data_final, output = 'markdown')
```

	n	media	DP	mediana	q25	q75	IQR
no	2538.00	30.44	7.27	30.00	25.00	35.00	10.00
yes	832.00	29.81	6.78	30.00	25.00	34.25	9.25

```
#t-test
t.test(age ~ vaccine, data = data_final)
```

```
##
##  Welch Two Sample t-test
##
## data:  age by vaccine
## t = 2.2691, df = 1506.4, p-value = 0.0234
## alternative hypothesis: true difference in means between group no and group yes is not equal to 0
## 95 percent confidence interval:
##  0.08480425 1.16655585
## sample estimates:
##  mean in group no mean in group yes
##          30.43578          29.81010
```

```
#effect size
c_cohen <- cohens_d(age ~ as.factor(vaccine), data=data_final)
c_cohen
```

```
## Cohen's d |       95% CI
## ------------------------
## 0.09      | [0.01, 0.17]
##
## - Estimated using pooled SD.
```

```
interpret_d(c_cohen$Cohens_d,rules="cohen1988")
```

```
## [1] "very small"
## (Rules: cohen1988)
```

Age group

```
with(data_final, ctable(age_group, vaccine, prop = "c", useNA = "no", chisq = TRUE, OR = TRUE))
```

```
## Cross-Tabulation, Column Proportions
## age_group * vaccine
## Data Frame: data_final
```

```
##
##
## ----------- --------- --------------- -------------- ---------------
##                vaccine              no            yes           Total
##   age_group
##         <20                169 (  6.7%)    63 (  7.6%)    232 (  6.9%)
##       20-34               1633 ( 64.3%)   561 ( 67.4%)   2194 ( 65.1%)
##        >=35                736 ( 29.0%)   208 ( 25.0%)    944 ( 28.0%)
##       Total               2538 (100.0%)   832 (100.0%)   3370 (100.0%)
## ----------- --------- --------------- -------------- ---------------
##
## ----------------------------
##  Chi.squared   df   p.value
## ------------- ---- ---------
##    5.2534      2    0.0723
## ----------------------------
```

Residence area

```
with(data_final, ctable(residence, vaccine, prop = "c", useNA = "no", chisq = FALSE, OR = TRUE))
```

```
## Cross-Tabulation, Column Proportions
## residence * vaccine
## Data Frame: data_final
##
## ----------- --------- --------------- -------------- ---------------
##                vaccine              no            yes           Total
##   residence
##   periurban                  8 (  0.3%)     2 (  0.3%)     10 (  0.3%)
##       rural                110 (  4.6%)    39 (  4.9%)    149 (  4.7%)
##       urban               2270 ( 95.1%)   749 ( 94.8%)   3019 ( 95.0%)
##       Total               2388 (100.0%)   790 (100.0%)   3178 (100.0%)
## ----------- --------- --------------- -------------- ---------------
```

```
fisher.test(data_final$residence, data_final$vaccine)
```

```
##
##  Fisher's Exact Test for Count Data
##
## data:  data_final$residence and data_final$vaccine
## p-value = 0.9147
## alternative hypothesis: two.sided
```

Comorbities

```
#Cardiac
data_final <-  data_final %>%
  mutate(cardiac = case_when(CARDIOPATI == 1 ~ "yes",
```

```
                             CARDIOPATI == 2 ~ "no",
                             TRUE ~ NA_character_))

#Hematologic
data_final <-  data_final %>%
  mutate(hematologic = case_when(HEMATOLOGI == 1 ~ "yes",
                                 HEMATOLOGI == 2 ~ "no",
                                 TRUE ~ NA_character_))

#Hepatic
data_final <-  data_final %>%
  mutate(hepatic = case_when(HEPATICA == 1 ~ "yes",
                             HEPATICA == 2 ~ "no",
                             TRUE ~ NA_character_))

#Asthma
data_final <-  data_final %>%
  mutate(asthma = case_when(ASMA == 1 ~ "yes",
                            ASMA == 2 ~ "no",
                            TRUE ~ NA_character_))

#Diabetes
data_final <-  data_final %>%
  mutate(diabetes = case_when(DIABETES == 1 ~ "yes",
                              DIABETES == 2 ~ "no",
                              TRUE ~ NA_character_))

#Neurologic
data_final <-  data_final %>%
  mutate(neurologic = case_when(NEUROLOGIC == 1 ~ "yes",
                                NEUROLOGIC == 2 ~ "no",
                                TRUE ~ NA_character_))

#Pneumologic
data_final <-  data_final %>%
  mutate(pneumologic = case_when(PNEUMOPATI == 1 ~ "yes",
                                 PNEUMOPATI == 2 ~ "no",
                                 TRUE ~ NA_character_))

#Imunossupression
data_final <-  data_final %>%
  mutate(imuno = case_when(IMUNODEPRE == 1 ~ "yes",
                           IMUNODEPRE == 2 ~ "no",
                           TRUE ~ NA_character_))

#Renal
data_final <-  data_final %>%
  mutate(renal = case_when(RENAL == 1 ~ "yes",
                           RENAL == 2 ~ "no",
                           TRUE ~ NA_character_))

#Obesity
data_final <-  data_final %>%
```

```r
  mutate(obesity = case_when(OBESIDADE == 1 ~ "yes",
                             OBESIDADE == 2 ~ "no",
                             TRUE ~ NA_character_))

#Any comorbidity

df <- data_final %>%
  select(cardiac,obesity,hematologic,hepatic,asthma,diabetes,neurologic,pneumologic,imuno,renal)

#if all comorbities in df are NA (not available), return NA.
soma <- function(x){
  if (sum(is.na(x))==10)
    return(NA_character_)
  else
    return(sum(!is.na(x) & x=="yes"))
}
data_final$qt_comorb_aux <- apply(df,1,soma)

data_final <- data_final %>%
  mutate(comorbidity = case_when(qt_comorb_aux >=1 ~ "yes",
                                 qt_comorb_aux ==0 ~ "no",
                                 TRUE ~ NA_character_))
```

Cardiac

```r
with(data_final, ctable(vaccine, cardiac, prop = "r", useNA = "no", chisq = TRUE, OR = TRUE))
```

```
## Cross-Tabulation, Row Proportions
## vaccine * cardiac
## Data Frame: data_final
##
##
## --------- --------- -------------- ------------- ---------------
##             cardiac             no           yes           Total
##   vaccine
##        no              994 (87.0%)   149 (13.0%)   1143 (100.0%)
##       yes              310 (83.6%)    61 (16.4%)    371 (100.0%)
##     Total             1304 (86.1%)   210 (13.9%)   1514 (100.0%)
## --------- --------- -------------- ------------- ---------------
##
## ----------------------------
##  Chi.squared   df   p.value
## ------------- ---- ---------
##    2.4424      1    0.1181
## ----------------------------
##
## ----------------------------------
##  Odds Ratio   Lo - 95%   Hi - 95%
## ------------ ---------- ----------
##     1.31        0.95       1.82
## ----------------------------------
```

Hematologic

```
with(data_final, ctable(vaccine, hematologic, prop = "r", useNA = "no", chisq = FALSE, OR = TRUE))
```

```
## Cross-Tabulation, Row Proportions
## vaccine * hematologic
## Data Frame: data_final
##
##
## --------- ------------- -------------- ----------- ---------------
##             hematologic             no         yes           Total
##   vaccine
##        no                 1108 (98.8%)   14 (1.2%)   1122 (100.0%)
##       yes                  353 (98.3%)    6 (1.7%)    359 (100.0%)
##     Total                 1461 (98.6%)   20 (1.4%)   1481 (100.0%)
## --------- ------------- -------------- ----------- ---------------
##
## ----------------------------------
##  Odds Ratio   Lo - 95%   Hi - 95%
## ------------ ---------- ----------
##     1.35        0.51       3.53
## ----------------------------------
```

```
fisher.test(data_final$hematologic, data_final$vaccine)
```

```
##
##  Fisher's Exact Test for Count Data
##
## data:  data_final$hematologic and data_final$vaccine
## p-value = 0.599
## alternative hypothesis: true odds ratio is not equal to 1
## 95 percent confidence interval:
##  0.4203391 3.7601885
## sample estimates:
## odds ratio
##   1.344915
```

Diabetes

```
ctable(data_final$vaccine, data_final$diabetes, chisq=TRUE, prop="r",  useNA = "no", OR = TRUE)
```

```
## Cross-Tabulation, Row Proportions
## vaccine * diabetes
## Data Frame: data_final
##
##
## --------- ---------- -------------- ------------- ---------------
##             diabetes             no           yes           Total
##   vaccine
```

```
##        no                995 (85.9%)   163 (14.1%)   1158 (100.0%)
##       yes                312 (84.1%)    59 (15.9%)    371 (100.0%)
##     Total               1307 (85.5%)   222 (14.5%)   1529 (100.0%)
## --------- ---------- -------------- ------------- ---------------
##
## ----------------------------
##  Chi.squared   df   p.value
## ------------- ---- ---------
##    0.6156      1    0.4327
## ----------------------------
##
## ----------------------------------
##  Odds Ratio   Lo - 95%   Hi - 95%
## ------------ ---------- ----------
##     1.15        0.84       1.60
## ----------------------------------
```

Obesity

```
ctable(data_final$vaccine, data_final$obesity, chisq=TRUE, prop="r",  useNA = "no", OR = TRUE)
```

```
## Cross-Tabulation, Row Proportions
## vaccine * obesity
## Data Frame: data_final
##
##
## --------- --------- -------------- ------------- ---------------
##             obesity             no           yes           Total
##   vaccine
##        no               1015 (88.6%)   130 (11.4%)   1145 (100.0%)
##       yes                318 (86.9%)    48 (13.1%)    366 (100.0%)
##     Total               1333 (88.2%)   178 (11.8%)   1511 (100.0%)
## --------- --------- -------------- ------------- ---------------
##
## ----------------------------
##  Chi.squared   df   p.value
## ------------- ---- ---------
##    0.6669      1    0.4141
## ----------------------------
##
## ----------------------------------
##  Odds Ratio   Lo - 95%   Hi - 95%
## ------------ ---------- ----------
##     1.18        0.83       1.68
## ----------------------------------
```

Asthma

```
ctable(data_final$vaccine, data_final$asthma, chisq=TRUE, prop="r",  useNA = "no", OR = TRUE)
```

```
## Cross-Tabulation, Row Proportions
## vaccine * asthma
## Data Frame: data_final
##
##
## --------- -------- -------------- ------------- ---------------
##             asthma             no           yes           Total
##   vaccine
##        no            1052 (92.7%)    83 ( 7.3%)   1135 (100.0%)
##       yes             323 (87.8%)    45 (12.2%)    368 (100.0%)
##     Total            1375 (91.5%)   128 ( 8.5%)   1503 (100.0%)
## --------- -------- -------------- ------------- ---------------
##
## ----------------------------
##  Chi.squared   df   p.value
## ------------- ---- ---------
##     7.999      1    0.0047
## ----------------------------
##
## ----------------------------------
##  Odds Ratio   Lo - 95%   Hi - 95%
## ------------ ---------- ----------
##     1.77        1.20       2.59
## ----------------------------------
```

Hepatic

```
ctable(data_final$vaccine, data_final$hepatic, chisq=FALSE, prop="r",  useNA = "no", OR = TRUE)
```

```
## Cross-Tabulation, Row Proportions
## vaccine * hepatic
## Data Frame: data_final
##
##
## --------- --------- --------------- ----------- ---------------
##             hepatic              no         yes           Total
##   vaccine
##        no             1101 ( 99.0%)   11 (1.0%)   1112 (100.0%)
##       yes              359 ( 99.7%)    1 (0.3%)    360 (100.0%)
##     Total             1460 ( 99.2%)   12 (0.8%)   1472 (100.0%)
## --------- --------- --------------- ----------- ---------------
##
## ----------------------------------
##  Odds Ratio   Lo - 95%   Hi - 95%
## ------------ ---------- ----------
##    0.279       0.036      2.167
## ----------------------------------
```

```
fisher.test(data_final$vaccine, data_final$hepatic)
```

```
##
```

```
##  Fisher's Exact Test for Count Data
##
## data:  data_final$vaccine and data_final$hepatic
## p-value = 0.3134
## alternative hypothesis: true odds ratio is not equal to 1
## 95 percent confidence interval:
##  0.006463293 1.931471269
## sample estimates:
## odds ratio
##  0.2789632
```

Neurologic

```
ctable(data_final$vaccine, data_final$neurologic, chisq=TRUE, prop="r",  useNA = "no", OR = TRUE)
```

```
## Cross-Tabulation, Row Proportions
## vaccine * neurologic
## Data Frame: data_final
##
##
## --------- ------------ -------------- ----------- ---------------
##             neurologic             no         yes           Total
##   vaccine
##        no                1102 (98.3%)   19 (1.7%)   1121 (100.0%)
##       yes                 352 (97.2%)   10 (2.8%)    362 (100.0%)
##     Total                1454 (98.0%)   29 (2.0%)   1483 (100.0%)
## --------- ------------ -------------- ----------- ---------------
##
## ----------------------------
##  Chi.squared   df   p.value
## ------------- ---- ---------
##    1.1173      1    0.2905
## ----------------------------
##
## ----------------------------------
##  Odds Ratio   Lo - 95%   Hi - 95%
## ------------ ---------- ----------
##     1.65        0.76       3.58
## ----------------------------------
```

Pneumologic

```
ctable(data_final$vaccine, data_final$pneumologic, chisq=TRUE, prop="r",  useNA = "no", OR = TRUE)
```

```
## Cross-Tabulation, Row Proportions
## vaccine * pneumologic
## Data Frame: data_final
##
##
```

```
## --------- ------------- -------------- ----------- ---------------
##            pneumologic             no         yes           Total
##   vaccine
##        no                 1103 (98.1%)   21 (1.9%)   1124 (100.0%)
##       yes                  353 (97.5%)    9 (2.5%)    362 (100.0%)
##     Total                 1456 (98.0%)   30 (2.0%)   1486 (100.0%)
## --------- ------------- -------------- ----------- ---------------
##
## ----------------------------
##  Chi.squared   df   p.value
## ------------- ---- ---------
##    0.2622      1    0.6086
## ----------------------------
##
## ----------------------------------
##  Odds Ratio   Lo - 95%   Hi - 95%
## ------------ ---------- ----------
##     1.34        0.61       2.95
## ----------------------------------
```

Imunossupression

```
ctable(data_final$vaccine, data_final$imuno, chisq=TRUE, prop="r", useNA = "no", OR = TRUE)
```

```
## Cross-Tabulation, Row Proportions
## vaccine * imuno
## Data Frame: data_final
##
##
## --------- ------- -------------- ----------- ---------------
##            imuno             no         yes           Total
##   vaccine
##        no           1083 (96.7%)   37 (3.3%)   1120 (100.0%)
##       yes            354 (98.3%)    6 (1.7%)    360 (100.0%)
##     Total           1437 (97.1%)   43 (2.9%)   1480 (100.0%)
## --------- ------- -------------- ----------- ---------------
##
## ----------------------------
##  Chi.squared   df   p.value
## ------------- ---- ---------
##    2.0399      1    0.1532
## ----------------------------
##
## ----------------------------------
##  Odds Ratio   Lo - 95%   Hi - 95%
## ------------ ---------- ----------
##     0.50        0.21       1.19
## ----------------------------------
```

Renal

```
ctable(data_final$vaccine, data_final$renal, chisq=FALSE, prop="r",  useNA = "no", OR = TRUE)
```

```
## Cross-Tabulation, Row Proportions
## vaccine * renal
## Data Frame: data_final
##
##
## --------- -------- -------------- ----------- ---------------
##              renal             no         yes           Total
##   vaccine
##        no           1100 (98.8%)   13 (1.2%)   1113 (100.0%)
##       yes            353 (98.6%)    5 (1.4%)    358 (100.0%)
##     Total           1453 (98.8%)   18 (1.2%)   1471 (100.0%)
## --------- -------- -------------- ----------- ---------------
##
## ----------------------------------
##  Odds Ratio   Lo - 95%   Hi - 95%
## ------------ ---------- ----------
##     1.20        0.42       3.39
## ----------------------------------
```

```
fisher.test(data_final$renal, data_final$vaccine)
```

```
##
##  Fisher's Exact Test for Count Data
##
## data:  data_final$renal and data_final$vaccine
## p-value = 0.7825
## alternative hypothesis: true odds ratio is not equal to 1
## 95 percent confidence interval:
##  0.332141 3.613119
## sample estimates:
## odds ratio
##   1.198399
```

Any comorbidity

```
with(data_final, ctable(vaccine, comorbidity, prop = "r", useNA = "no", chisq = TRUE, OR = TRUE))
```

```
## Cross-Tabulation, Row Proportions
## vaccine * comorbidity
## Data Frame: data_final
##
##
## --------- ------------- ------------- ------------- ---------------
##              comorbidity            no           yes           Total
##   vaccine
```

```
##        no                  748 (60.3%)   492 (39.7%)   1240 (100.0%)
##       yes                  210 (52.4%)   191 (47.6%)    401 (100.0%)
##     Total                  958 (58.4%)   683 (41.6%)   1641 (100.0%)
## --------- ------------- ------------- ------------- ---------------
##
## ----------------------------
##  Chi.squared   df   p.value
## ------------- ---- ---------
##    7.5648      1     0.006
## ----------------------------
##
## ----------------------------------
##  Odds Ratio   Lo - 95%   Hi - 95%
## ------------ ---------- ----------
##     1.38        1.10       1.73
## ----------------------------------
```

Symptoms

```
# Fever
data_final <-  data_final %>%
  mutate(fever = case_when(FEBRE == 1 ~ "yes",
                           FEBRE == 2 ~ "no",
                           TRUE ~ NA_character_))

# Cough
data_final <-  data_final %>%
  mutate(cough = case_when(TOSSE == 1 ~ "yes",
                           TOSSE == 2 ~ "no",
                           TRUE ~ NA_character_))

# Sore throat
data_final <-  data_final %>%
  mutate(sore_throat = case_when(GARGANTA == 1 ~ "yes",
                                 GARGANTA == 2 ~ "no",
                                 TRUE ~ NA_character_))

# Dyspnea
data_final <-  data_final %>%
  mutate(dyspnea = case_when(DISPNEIA == 1 ~ "yes",
                             DISPNEIA == 2 ~ "no",
                             TRUE ~ NA_character_))

# Respiratory discomfort
data_final <-  data_final %>%
  mutate(resp_disc = case_when(DESC_RESP == 1 ~ "yes",
                               DESC_RESP == 2 ~ "no",
                               TRUE ~ NA_character_))

# Desaturation
data_final <-  data_final %>%
```

```r
  mutate(desaturation = case_when(SATURACAO == 1 ~ "yes",
                                  SATURACAO == 2 ~ "no",
                                  TRUE ~ NA_character_))

# Diarrhea
data_final <-  data_final %>%
  mutate(diarrhea = case_when(DIARREIA == 1 ~ "yes",
                              DIARREIA == 2 ~ "no",
                              TRUE ~ NA_character_))

# Vomit
data_final <-  data_final %>%
  mutate(vomit = case_when(VOMITO == 1 ~ "yes",
                           VOMITO == 2 ~ "no",
                           TRUE ~ NA_character_))

# Abdominal pain
data_final <-  data_final %>%
  mutate(abd_pain = case_when(DOR_ABD == 1 ~ "yes",
                              DOR_ABD == 2 ~ "no",
                              TRUE ~ NA_character_))

# Fatigue
data_final <-  data_final %>%
  mutate(fatigue = case_when(FADIGA == 1 ~ "yes",
                             FADIGA == 2 ~ "no",
                             TRUE ~ NA_character_))

# Olfactory loss
data_final <-  data_final %>%
  mutate(olfac_loss = case_when(PERD_OLFT == 1 ~ "yes",
                                PERD_OLFT == 2 ~ "no",
                                TRUE ~ NA_character_))

# Loss of taste
data_final <-  data_final %>%
  mutate(loss_taste = case_when(PERD_PALA == 1 ~ "yes",
                                PERD_PALA == 2 ~ "no",
                                TRUE ~ NA_character_))

# Any respiratory symptom
df <- data_final %>%
  select(dyspnea,fatigue,desaturation,resp_disc)

soma <- function(x){
  if (sum(is.na(x))==4)
    return(NA_character_)
  else
    return(sum(!is.na(x) & x=="yes"))
}
data_final$qt_sintomas_resp_aux <- apply(df,1,soma)

data_final <-  data_final %>%
```

```r
  mutate(resp_symp = case_when(qt_sintomas_resp_aux >=1 ~ "yes",
                               qt_sintomas_resp_aux ==0 ~ "no",
                               TRUE ~ NA_character_))
# Any symptom
df <- data_final %>%
  select(dyspnea,fatigue,desaturation,resp_disc,
         fever,cough,sore_throat,diarrhea,vomit,abd_pain,olfac_loss,loss_taste)
soma <- function(x){
  if (sum(is.na(x))==12)
    return(NA_character_)
  else
    return(sum(!is.na(x) & x=="yes"))
}
data_final$qt_sintomas_aux <- apply(df,1,soma)

data_final <-  data_final %>%
  mutate(symptom = case_when(qt_sintomas_aux >= 1 ~ "yes",
                             qt_sintomas_aux == 0 ~ "no",
                             TRUE ~ NA_character_))
```

Fever

```r
with(data_final, ctable(vaccine, fever, prop = "r", useNA = "no", chisq = TRUE, OR=TRUE))
```

```
## Cross-Tabulation, Row Proportions
## vaccine * fever
## Data Frame: data_final
##
##
## --------- ------- -------------- -------------- ---------------
##             fever             no            yes           Total
##   vaccine
##        no            842 (35.5%)   1529 (64.5%)   2371 (100.0%)
##       yes            255 (33.4%)    509 (66.6%)    764 (100.0%)
##     Total           1097 (35.0%)   2038 (65.0%)   3135 (100.0%)
## --------- ------- -------------- -------------- ---------------
##
## ----------------------------
##  Chi.squared   df   p.value
## ------------- ---- ---------
##    1.0664      1    0.3018
## ----------------------------
##
## ----------------------------------
##  Odds Ratio    Lo - 95%    Hi - 95%
## ------------ ----------- ----------
##     1.10        0.93        1.31
## ----------------------------------
```

Cough

```r
with(data_final, ctable(vaccine, cough, prop = "r", useNA = "no", chisq = TRUE, OR=TRUE))
```

```
## Cross-Tabulation, Row Proportions
## vaccine * cough
## Data Frame: data_final
##
##
## --------- ------- ------------- -------------- ---------------
##             cough            no            yes           Total
##   vaccine
##        no           594 (24.7%)   1808 (75.3%)   2402 (100.0%)
##       yes           189 (24.2%)    593 (75.8%)    782 (100.0%)
##     Total           783 (24.6%)   2401 (75.4%)   3184 (100.0%)
## --------- ------- ------------- -------------- ---------------
##
## ----------------------------
##  Chi.squared   df   p.value
## ------------- ---- ---------
##     0.072      1    0.7884
## ----------------------------
##
## ----------------------------------
##  Odds Ratio   Lo - 95%   Hi - 95%
## ------------ ---------- ----------
##     1.03        0.85       1.24
## ----------------------------------
```

Sore throat

```r
with(data_final, ctable(vaccine, sore_throat, prop = "r", useNA = "no", chisq = TRUE, OR=TRUE))
```

```
## Cross-Tabulation, Row Proportions
## vaccine * sore_throat
## Data Frame: data_final
##
##
## --------- ------------- -------------- ------------- ---------------
##             sore_throat             no           yes           Total
##   vaccine
##        no                 1524 (69.7%)   664 (30.3%)   2188 (100.0%)
##       yes                  494 (68.0%)   233 (32.0%)    727 (100.0%)
##     Total                 2018 (69.2%)   897 (30.8%)   2915 (100.0%)
## --------- ------------- -------------- ------------- ---------------
##
## ----------------------------
##  Chi.squared   df   p.value
## ------------- ---- ---------
##    0.6644      1     0.415
```

```
## ----------------------------
##
## ----------------------------------
##  Odds Ratio   Lo - 95%   Hi - 95%
## ------------ ---------- ----------
##     1.08        0.90       1.30
## ----------------------------------
```

Dyspnea

```
with(data_final, ctable(vaccine, dyspnea, prop = "r", useNA = "no", chisq = TRUE, OR=TRUE))
```

```
## Cross-Tabulation, Row Proportions
## vaccine * dyspnea
## Data Frame: data_final
##
##
## --------- --------- -------------- -------------- ---------------
##             dyspnea             no            yes           Total
##   vaccine
##        no               881 (37.4%)   1477 (62.6%)   2358 (100.0%)
##       yes               341 (44.9%)    418 (55.1%)    759 (100.0%)
##     Total              1222 (39.2%)   1895 (60.8%)   3117 (100.0%)
## --------- --------- -------------- -------------- ---------------
##
## ----------------------------
##  Chi.squared   df   p.value
## ------------- ---- ---------
##    13.4724     1     2e-04
## ----------------------------
##
## ----------------------------------
##  Odds Ratio   Lo - 95%   Hi - 95%
## ------------ ---------- ----------
##     0.73        0.62       0.86
## ----------------------------------
```

Respiratory discomfort

```
with(data_final, ctable(vaccine, resp_disc, prop = "r", useNA = "no", chisq = TRUE, OR=TRUE))
```

```
## Cross-Tabulation, Row Proportions
## vaccine * resp_disc
## Data Frame: data_final
##
##
## --------- ----------- -------------- -------------- ---------------
##             resp_disc             no            yes           Total
##   vaccine
```

```
##        no                1071 (47.2%)   1198 (52.8%)   2269 (100.0%)
##       yes                 337 (45.1%)    411 (54.9%)    748 (100.0%)
##     Total                1408 (46.7%)   1609 (53.3%)   3017 (100.0%)
## --------- ----------- -------------- -------------- ---------------
##
## ----------------------------
##  Chi.squared   df   p.value
## ------------- ---- ---------
##    0.9583      1    0.3276
## ----------------------------
##
## ----------------------------------
##  Odds Ratio   Lo - 95%   Hi - 95%
## ------------ ---------- ----------
##     1.09        0.92       1.29
## ----------------------------------
```

Desaturation

```
with(data_final, ctable(vaccine, desaturation, prop = "r", useNA = "no", chisq = TRUE, OR=TRUE))
```

```
## Cross-Tabulation, Row Proportions
## vaccine * desaturation
## Data Frame: data_final
##
##
## --------- -------------- -------------- -------------- ---------------
##              desaturation             no            yes           Total
##   vaccine
##        no                   1316 (58.4%)    938 (41.6%)   2254 (100.0%)
##       yes                    491 (66.9%)    243 (33.1%)    734 (100.0%)
##     Total                   1807 (60.5%)   1181 (39.5%)   2988 (100.0%)
## --------- -------------- -------------- -------------- ---------------
##
## ----------------------------
##  Chi.squared   df   p.value
## ------------- ---- ---------
##    16.4163     1     1e-04
## ----------------------------
##
## ----------------------------------
##  Odds Ratio   Lo - 95%   Hi - 95%
## ------------ ---------- ----------
##     0.69        0.58       0.83
## ----------------------------------
```

Diarrhea

```
with(data_final, ctable(vaccine, diarrhea, prop = "r", useNA = "no", chisq = TRUE, OR=TRUE))
```

```
## Cross-Tabulation, Row Proportions
## vaccine * diarrhea
## Data Frame: data_final
##
##
## --------- ---------- -------------- ------------- ---------------
##             diarrhea             no           yes           Total
##   vaccine
##        no               1801 (84.6%)   329 (15.4%)   2130 (100.0%)
##       yes                595 (83.1%)   121 (16.9%)    716 (100.0%)
##     Total               2396 (84.2%)   450 (15.8%)   2846 (100.0%)
## --------- ---------- -------------- ------------- ---------------
##
## ----------------------------
##  Chi.squared   df   p.value
## ------------- ---- ---------
##    0.7447      1    0.3882
## ----------------------------
##
## ----------------------------------
##  Odds Ratio   Lo - 95%   Hi - 95%
## ------------ ---------- ----------
##     1.11        0.89       1.40
## ----------------------------------
```

Vomit

```r
with(data_final, ctable(vaccine, vomit, prop = "r", useNA = "no", chisq = TRUE, OR=TRUE))
```

```
## Cross-Tabulation, Row Proportions
## vaccine * vomit
## Data Frame: data_final
##
##
## --------- ------- -------------- ------------- ---------------
##             vomit             no           yes           Total
##   vaccine
##        no            1833 (85.8%)   303 (14.2%)   2136 (100.0%)
##       yes             609 (84.8%)   109 (15.2%)    718 (100.0%)
##     Total            2442 (85.6%)   412 (14.4%)   2854 (100.0%)
## --------- ------- -------------- ------------- ---------------
##
## ----------------------------
##  Chi.squared   df   p.value
## ------------- ---- ---------
##    0.3544      1    0.5516
## ----------------------------
##
## ----------------------------------
##  Odds Ratio   Lo - 95%   Hi - 95%
## ------------ ---------- ----------
##     1.08        0.85       1.37
```

```
## ----------------------------------
```

Abdominal pain

```r
with(data_final, ctable(vaccine, abd_pain, prop = "r", useNA = "no", chisq = TRUE, OR=TRUE))
```

```
## Cross-Tabulation, Row Proportions
## vaccine * abd_pain
## Data Frame: data_final
##
##
## --------- ---------- -------------- ------------- ---------------
##             abd_pain             no           yes           Total
##   vaccine
##        no              1410 (88.3%)   187 (11.7%)   1597 (100.0%)
##       yes               384 (87.3%)    56 (12.7%)    440 (100.0%)
##     Total              1794 (88.1%)   243 (11.9%)   2037 (100.0%)
## --------- ---------- -------------- ------------- ---------------
##
## ----------------------------
##  Chi.squared   df   p.value
## ------------- ---- ---------
##    0.2502      1     0.617
## ----------------------------
##
## ----------------------------------
##  Odds Ratio   Lo - 95%   Hi - 95%
## ------------ ---------- ----------
##     1.10        0.80       1.51
## ----------------------------------
```

Fatigue

```r
with(data_final, ctable(vaccine, fatigue, prop = "r", useNA = "no", chisq = TRUE, OR=TRUE))
```

```
## Cross-Tabulation, Row Proportions
## vaccine * fatigue
## Data Frame: data_final
##
##
## --------- --------- -------------- ------------- ---------------
##             fatigue             no           yes           Total
##   vaccine
##        no             1117 (68.4%)   516 (31.6%)   1633 (100.0%)
##       yes              290 (64.4%)   160 (35.6%)    450 (100.0%)
##     Total             1407 (67.5%)   676 (32.5%)   2083 (100.0%)
## --------- --------- -------------- ------------- ---------------
##
## ----------------------------
```

```
##   Chi.squared   df   p.value
## ------------- ---- ---------
##    2.3429      1    0.1259
## ----------------------------
##
## ----------------------------------
##  Odds Ratio   Lo - 95%   Hi - 95%
## ------------ ---------- ----------
##     1.19        0.96       1.49
## ----------------------------------
```

Olfactory loss

```r
with(data_final, ctable(vaccine, olfac_loss, prop = "r", useNA = "no", chisq = TRUE, OR=TRUE))
```

```
## Cross-Tabulation, Row Proportions
## vaccine * olfac_loss
## Data Frame: data_final
##
##
## --------- ------------ -------------- ------------- ---------------
##             olfac_loss             no           yes           Total
##   vaccine
##        no               1288 (79.1%)   340 (20.9%)   1628 (100.0%)
##       yes                320 (68.8%)   145 (31.2%)    465 (100.0%)
##     Total               1608 (76.8%)   485 (23.2%)   2093 (100.0%)
## --------- ------------ -------------- ------------- ---------------
##
## ----------------------------
##  Chi.squared   df   p.value
## ------------- ---- ---------
##    20.972      1       0
## ----------------------------
##
## ----------------------------------
##  Odds Ratio   Lo - 95%   Hi - 95%
## ------------ ---------- ----------
##     1.72        1.36       2.16
## ----------------------------------
```

Loss of taste

```r
with(data_final, ctable(vaccine, loss_taste, prop = "r", useNA = "no", chisq = TRUE, OR=TRUE))
```

```
## Cross-Tabulation, Row Proportions
## vaccine * loss_taste
## Data Frame: data_final
##
##
```

```
## --------- ------------ -------------- ------------- ---------------
##            loss_taste             no           yes           Total
##   vaccine                                                        
##        no                1302 (79.8%)   329 (20.2%)   1631 (100.0%)
##       yes                 324 (70.3%)   137 (29.7%)    461 (100.0%)
##     Total                1626 (77.7%)   466 (22.3%)   2092 (100.0%)
## --------- ------------ -------------- ------------- ---------------
## 
## ----------------------------
##  Chi.squared   df   p.value 
## ------------- ---- ---------
##    18.371      1       0    
## ----------------------------
## 
## ----------------------------------
##  Odds Ratio   Lo - 95%   Hi - 95% 
## ------------ ---------- ----------
##     1.67        1.32       2.11   
## ----------------------------------
```

Any respiratory symptom

```r
with(data_final, ctable(vaccine, resp_symp, prop = "r", useNA = "no", chisq = TRUE, OR=TRUE))
```

```
## Cross-Tabulation, Row Proportions
## vaccine * resp_symp
## Data Frame: data_final
## 
## 
## --------- ----------- ------------- -------------- ---------------
##            resp_symp            no            yes           Total
##   vaccine                                                        
##        no               562 (23.2%)   1865 (76.8%)   2427 (100.0%)
##       yes               213 (27.2%)    570 (72.8%)    783 (100.0%)
##     Total               775 (24.1%)   2435 (75.9%)   3210 (100.0%)
## --------- ----------- ------------- -------------- ---------------
## 
## ----------------------------
##  Chi.squared   df   p.value 
## ------------- ---- ---------
##    5.0753      1    0.0243  
## ----------------------------
## 
## ----------------------------------
##  Odds Ratio   Lo - 95%   Hi - 95% 
## ------------ ---------- ----------
##     0.81        0.67       0.97   
## ----------------------------------
```

Any symptom

```
with(data_final, ctable(vaccine, symptom, prop = "r", useNA = "no", chisq = TRUE, OR=TRUE))
```

```
## Cross-Tabulation, Row Proportions
## vaccine * symptom
## Data Frame: data_final
##
##
## --------- --------- ------------ -------------- ---------------
##             symptom           no            yes           Total
##   vaccine
##        no              102 (4.1%)   2415 (95.9%)   2517 (100.0%)
##       yes               35 (4.3%)    781 (95.7%)    816 (100.0%)
##     Total              137 (4.1%)   3196 (95.9%)   3333 (100.0%)
## --------- --------- ------------ -------------- ---------------
##
## ----------------------------
##  Chi.squared   df   p.value
## ------------- ---- ---------
##    0.0379      1    0.8457
## ----------------------------
##
## ----------------------------------
##  Odds Ratio   Lo - 95%   Hi - 95%
## ------------ ---------- ----------
##     0.94        0.64       1.40
## ----------------------------------
```

Outcome

```
# Hospitalization
data_final <- data_final %>%
  mutate(hospitalization = case_when(HOSPITAL == 1 ~ "yes",
                         HOSPITAL == 2 ~ "no",
                         TRUE ~ NA_character_))

# ICU
data_final <- data_final %>%
  mutate(icu = case_when(UTI == 1 ~ "yes",
                         UTI == 2 ~ "no",
                         TRUE ~ NA_character_))


# Length time in ICU
data_final<-  data_final%>%
  mutate(dt_enticu  = as.Date(DT_ENTUTI,  format = "%d/%m/%Y"),
         dt_exicu = as.Date(DT_SAIDUTI, format = "%d/%m/%Y"),
         time_icu = as.numeric(dt_exicu - dt_enticu)
  )
```

```r
# ventilatory support
data_final <- data_final %>%
  mutate(ventilatory_support = case_when(SUPORT_VEN == 1 ~ "invasive",
                              SUPORT_VEN == 2 ~ "non-invasive",
                                SUPORT_VEN == 3 ~ "no",
                              TRUE ~ NA_character_))

# Intubation
data_final <- data_final %>%
  mutate(intubation = case_when(SUPORT_VEN == 1 ~ "yes",
                              SUPORT_VEN == 2 | SUPORT_VEN == 3 ~ "no",
                              TRUE ~ NA_character_))
```

Hospitalization

```r
with(data_final, ctable(vaccine, hospitalization, prop = "r", useNA = "no", chisq = TRUE, OR=TRUE))
```

```
## Cross-Tabulation, Row Proportions
## vaccine * hospitalization
## Data Frame: data_final
##
##
## --------- ----------------- ------------ -------------- ---------------
##             hospitalization           no            yes           Total
##   vaccine
##        no                     70 (2.8%)   2454 (97.2%)   2524 (100.0%)
##       yes                     35 (4.2%)    789 (95.8%)    824 (100.0%)
##     Total                    105 (3.1%)   3243 (96.9%)   3348 (100.0%)
## --------- ----------------- ------------ -------------- ---------------
##
## ----------------------------
##  Chi.squared   df   p.value
## ------------- ---- ---------
##     3.972      1    0.0463
## ----------------------------
##
## ----------------------------------
##  Odds Ratio   Lo - 95%   Hi - 95%
## ------------ ---------- ----------
##     0.64        0.43       0.97
## ----------------------------------
```

ICU

```r
with(data_final, ctable(vaccine, icu, prop = "r", useNA = "no", chisq = TRUE, OR=TRUE))
```

```
## Cross-Tabulation, Row Proportions
## vaccine * icu
```

```
## Data Frame: data_final
##
##
## --------- ----- -------------- ------------- ---------------
##              icu             no           yes           Total
##   vaccine
##        no         1733 (73.4%)   629 (26.6%)   2362 (100.0%)
##       yes          597 (78.9%)   160 (21.1%)    757 (100.0%)
##     Total         2330 (74.7%)   789 (25.3%)   3119 (100.0%)
## --------- ----- -------------- ------------- ---------------
##
## ----------------------------
##  Chi.squared   df   p.value
## ------------- ---- ---------
##    8.8679      1    0.0029
## ----------------------------
##
## ----------------------------------
##  Odds Ratio   Lo - 95%   Hi - 95%
## ------------ ---------- ----------
##     0.74        0.61       0.90
## ----------------------------------
```

Length time in ICU

```
data_final_aux <- data_final %>%
  filter (icu == "yes")
```

```
datasummary((vaccine) ~ time_icu*(n+media+DP+mediana+q25+q75+IQR),
            data = data_final_aux, output = 'markdown')
```

	n	media	DP	mediana	q25	q75	IQR
no	399.00	12.15	12.63	8.00	4.00	17.00	13.00
yes	110.00	11.83	11.25	8.00	4.00	17.00	13.00

```
#t-test
t.test(time_icu ~ vaccine, data = data_final_aux)
```

```
##
##  Welch Two Sample t-test
##
## data:  time_icu by vaccine
## t = 0.25552, df = 191.67, p-value = 0.7986
## alternative hypothesis: true difference in means between group no and group yes is not equal to 0
## 95 percent confidence interval:
##  -2.137340  2.773521
## sample estimates:
##  mean in group no mean in group yes
##          12.14536          11.82727
```

Ventilatory support

```
with(data_final, ctable(vaccine, ventilatory_support, prop = "r", useNA = "no", chisq = TRUE, OR=TRUE))
```

```
## Cross-Tabulation, Row Proportions
## vaccine * ventilatory_support
## Data Frame: data_final
##
##
## --------- --------------------- ------------- -------------- -------------- ---------------
##           ventilatory_support      invasive             no   non-invasive           Total
##   vaccine
##        no                       336 (14.3%)   1192 (50.6%)    827 (35.1%)   2355 (100.0%)
##       yes                        68 ( 8.8%)    450 (58.5%)    251 (32.6%)    769 (100.0%)
##     Total                       404 (12.9%)   1642 (52.6%)   1078 (34.5%)   3124 (100.0%)
## --------- --------------------- ------------- -------------- -------------- ---------------
##
## ----------------------------
##  Chi.squared   df   p.value
## ------------- ---- ---------
##    21.1094     2       0
## ----------------------------
```

Intubation

```
with(data_final, ctable(vaccine, intubation, prop = "r", useNA = "no", chisq = TRUE, OR=TRUE))
```

```
## Cross-Tabulation, Row Proportions
## vaccine * intubation
## Data Frame: data_final
##
##
## --------- ------------ -------------- ------------- ---------------
##           intubation             no           yes           Total
##   vaccine
##        no               2019 (85.7%)   336 (14.3%)   2355 (100.0%)
##       yes                701 (91.2%)    68 ( 8.8%)    769 (100.0%)
##     Total               2720 (87.1%)   404 (12.9%)   3124 (100.0%)
## --------- ------------ -------------- ------------- ---------------
##
## ----------------------------
##  Chi.squared   df   p.value
## ------------- ---- ---------
##    14.6735     1     1e-04
## ----------------------------
##
## ----------------------------------
##  Odds Ratio   Lo - 95%   Hi - 95%
## ------------ ---------- ----------
##     0.58        0.44       0.77
## ----------------------------------
```

Death

```
with(data_final, ctable(vaccine, death, prop = "r", useNA = "no", chisq = TRUE, OR=TRUE))
```

```
## Cross-Tabulation, Row Proportions
## vaccine * death
## Data Frame: data_final
##
##
## --------- ------- -------------- ------------- ---------------
##             death           cure         death           Total
##   vaccine
##        no           2213 (87.2%)   325 (12.8%)   2538 (100.0%)
##       yes            788 (94.7%)    44 ( 5.3%)    832 (100.0%)
##     Total           3001 (89.1%)   369 (10.9%)   3370 (100.0%)
## --------- ------- -------------- ------------- ---------------
##
## ----------------------------
##  Chi.squared   df   p.value
## ------------- ---- ---------
##    35.5435     1       0
## ----------------------------
##
## ----------------------------------
##  Odds Ratio   Lo - 95%   Hi - 95%
## ------------ ---------- ----------
##     0.38        0.27       0.53
## ----------------------------------
```

Time between covid vaccine and first symptoms of covid-19

Selecting only the vaccinated cases

```
data_final_aux <- data_final %>%
  filter (vaccine == "yes")

data_final_aux$death <-
  factor(data_final_aux$death, levels = c("cure", "death"), label =  c("cure", "death"))
```

```
datasummary((death) ~ time_vaccine_symptoms*(n+media+DP+mediana+q25+q75+IQR),
            data = data_final_aux, output = 'markdown')
```

	n	media	DP	mediana	q25	q75	IQR
cure	788.00	118.11	104.17	85.50	31.00	184.00	153.00
death	44.00	129.57	136.86	63.50	43.50	212.50	169.00

```
#t-test
t.test(time_vaccine_symptoms ~ death, data = data_final_aux)
```

```
##
##  Welch Two Sample t-test
##
## data:  time_vaccine_symptoms by death
## t = -0.54639, df = 45.824, p-value = 0.5875
## alternative hypothesis: true difference in means between group cure and group death is not equal to 
## 95 percent confidence interval:
##  -53.65500  30.74707
## sample estimates:
##  mean in group cure mean in group death
##            118.1142            129.5682
```

Propensity Scoring Matching (PSM)

```
data_final <- data_final %>%
  mutate(vaccine1 = ifelse(vaccine == "yes",1,0),
         id = 1:dim(data_final)[1])

data_final1 <- data_final %>%
  select(id, vaccine1, white_color, age, education2, asthma)

data_final1 <- data_final1 %>%
  mutate(
    white_color1 = ifelse(is.na(white_color) == TRUE, "na", white_color),
    education21 = ifelse(is.na(education2) == TRUE, "na", education2),
    asthma1 = ifelse(is.na(asthma) == TRUE, "na", asthma)
  )

#PSM -  method = "nearest", ratio = 1
psm1 <- matchit(vaccine1 ~ white_color1 + age + education21 + asthma1, data = data_final1, method = "ne

summary(psm1)
```

```
##
## Call:
## matchit(formula = vaccine1 ~ white_color1 + age + education21 +
##     asthma1, data = data_final1, method = "nearest", ratio = 1)
##
## Summary of Balance for All Data:
##                 Means Treated Means Control Std. Mean Diff. Var. Ratio
## distance               0.2681        0.2399          0.3760     1.1378
## white_color1na         0.0709        0.0906         -0.0768          .
## white_color1no         0.4639        0.5165         -0.1055          .
## white_color1yes        0.4651        0.3928          0.1450          .
## age                   29.8101       30.4358         -0.0923     0.8685
## education211           0.1238        0.1383         -0.0440          .
## education212           0.4026        0.3176          0.1735          .
## education213           0.1671        0.0981          0.1849          .
## education21na          0.3065        0.4460         -0.3026          .
## asthma1na              0.5577        0.5528          0.0099          .
## asthma1no              0.3882        0.4145         -0.0539          .
```

```
## asthma1yes             0.0541        0.0327          0.0945          .
##                 eCDF Mean eCDF Max
## distance           0.1047   0.1801
## white_color1na     0.0197   0.0197
## white_color1no     0.0526   0.0526
## white_color1yes    0.0723   0.0723
## age                0.0144   0.0444
## education211       0.0145   0.0145
## education212       0.0851   0.0851
## education213       0.0690   0.0690
## education21na      0.1395   0.1395
## asthma1na          0.0049   0.0049
## asthma1no          0.0263   0.0263
## asthma1yes         0.0214   0.0214
##
##
## Summary of Balance for Matched Data:
##                 Means Treated Means Control Std. Mean Diff. Var. Ratio
## distance               0.2681        0.2679          0.0017     1.0072
## white_color1na         0.0709        0.0481          0.0890          .
## white_color1no         0.4639        0.4784         -0.0289          .
## white_color1yes        0.4651        0.4736         -0.0169          .
## age                   29.8101       29.7488          0.0090     1.0768
## education211           0.1238        0.1094          0.0438          .
## education212           0.4026        0.4123         -0.0196          .
## education213           0.1671        0.1647          0.0064          .
## education21na          0.3065        0.3137         -0.0156          .
## asthma1na              0.5577        0.5745         -0.0339          .
## asthma1no              0.3882        0.3774          0.0222          .
## asthma1yes             0.0541        0.0481          0.0266          .
##                 eCDF Mean eCDF Max Std. Pair Dist.
## distance           0.0003   0.0072          0.0024
## white_color1na     0.0228   0.0228          0.1639
## white_color1no     0.0144   0.0144          0.1446
## white_color1yes    0.0084   0.0084          0.1615
## age                0.0047   0.0180          0.2598
## education211       0.0144   0.0144          0.1241
## education212       0.0096   0.0096          0.1568
## education213       0.0024   0.0024          0.1740
## education21na      0.0072   0.0072          0.0626
## asthma1na          0.0168   0.0168          0.2226
## asthma1no          0.0108   0.0108          0.2195
## asthma1yes         0.0060   0.0060          0.1647
##
## Percent Balance Improvement:
##                 Std. Mean Diff. Var. Ratio eCDF Mean eCDF Max
## distance                   99.5       94.5      99.7     96.0
## white_color1na            -15.9          .     -15.9    -15.9
## white_color1no             72.6          .      72.6     72.6
## white_color1yes            88.4          .      88.4     88.4
## age                        90.2       47.5      67.1     59.4
## education211                0.5          .       0.5      0.5
## education212               88.7          .      88.7     88.7
## education213               96.5          .      96.5     96.5
```

```
## education21na                 94.8          .       94.8     94.8
## asthma1na                    -243.8          .     -243.8   -243.8
## asthma1no                      58.8          .       58.8     58.8
## asthma1yes                     71.9          .       71.9     71.9
##
## Sample Sizes:
##           Control Treated
## All          2538     832
## Matched       832     832
## Unmatched    1706       0
## Discarded       0       0
```

```
plot(psm1, type = "jitter", interactive = FALSE)
```

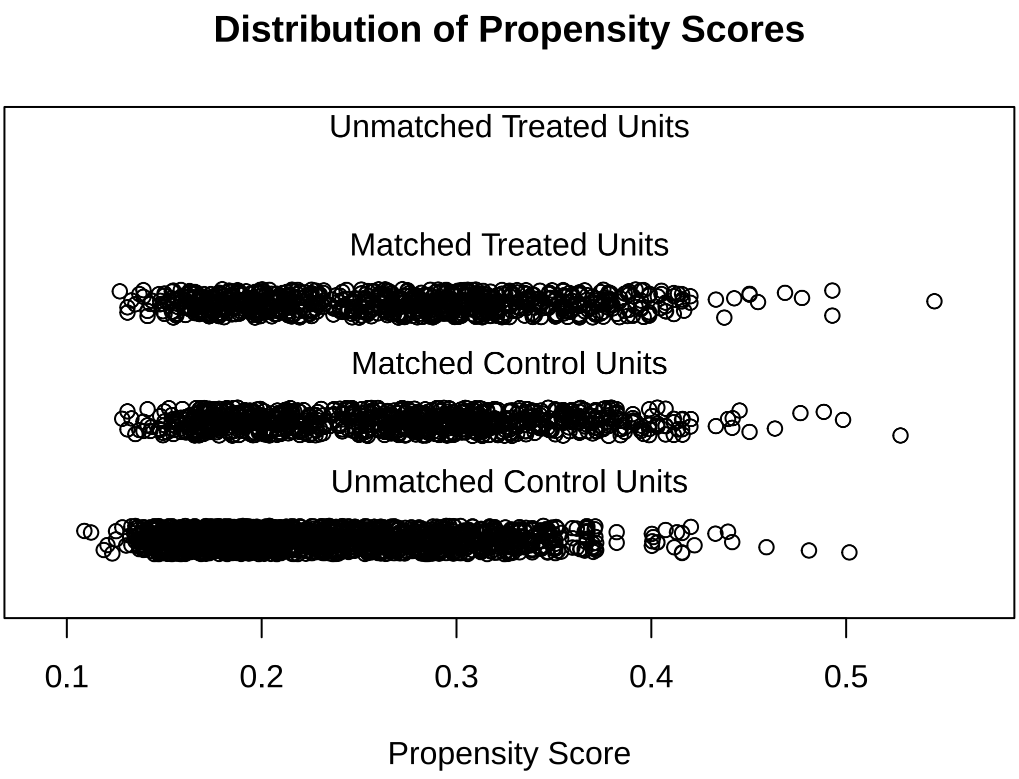


```
plot(psm1, type = "qq", interactive = FALSE,
     which.xs = c("white_color1","age","education21","asthma1"))
```

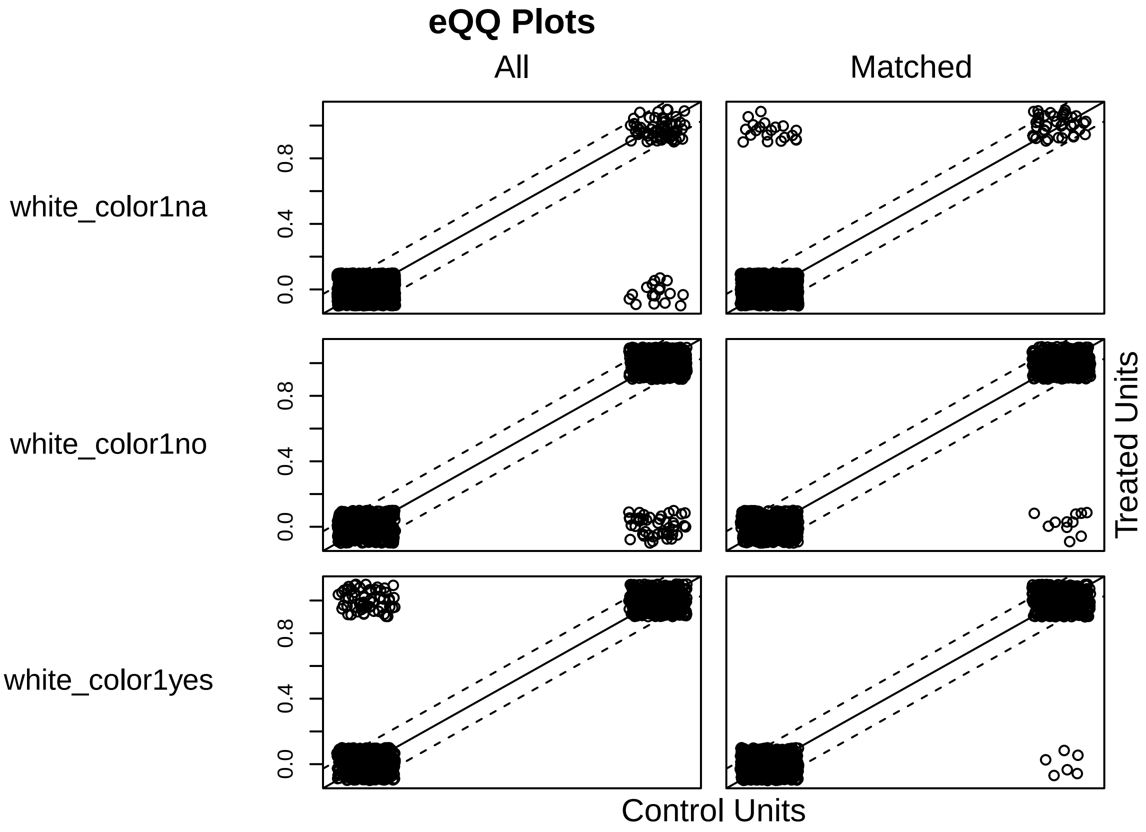
eQQ Plots
All
Matched
white_color1na
white_color1no
white_color1yes
0.0
0.4
0.8
Treated Units
Control Units

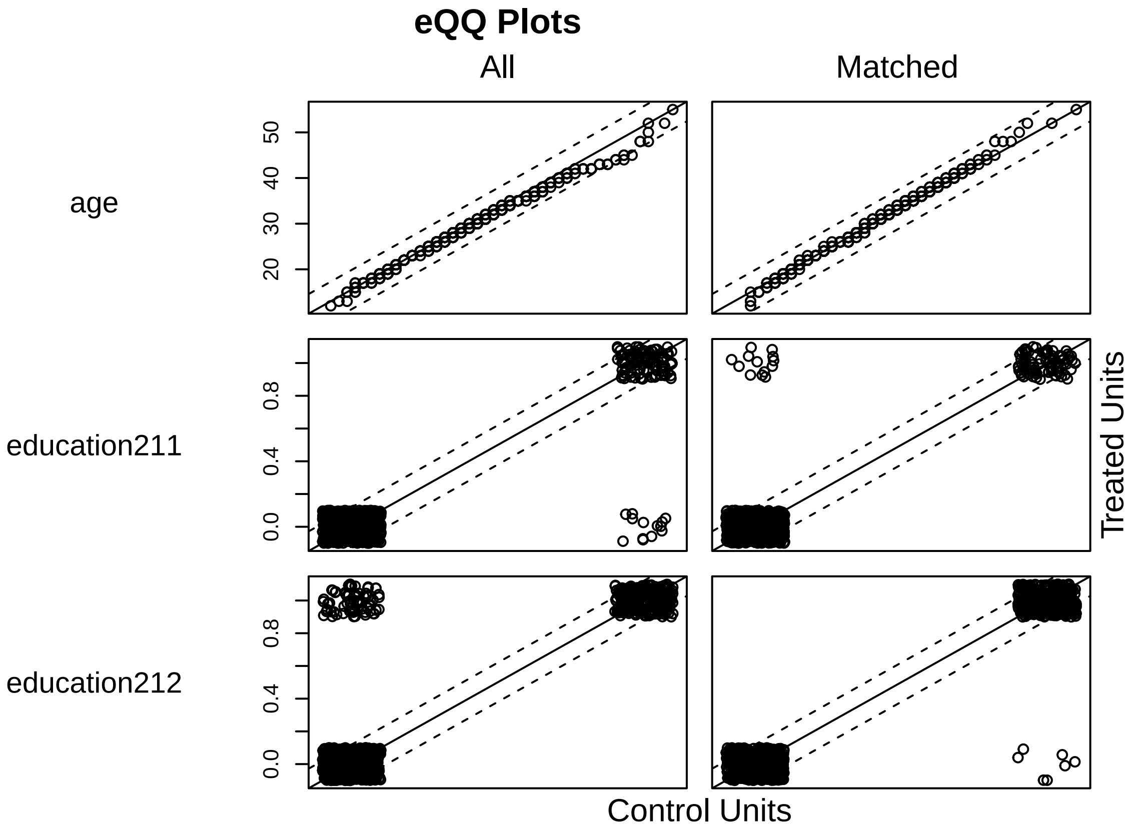

eQQ Plots
All
Matched
age
education211
education212
Treated Units
Control Units
50
40
30
20
0.8
0.4
0.0

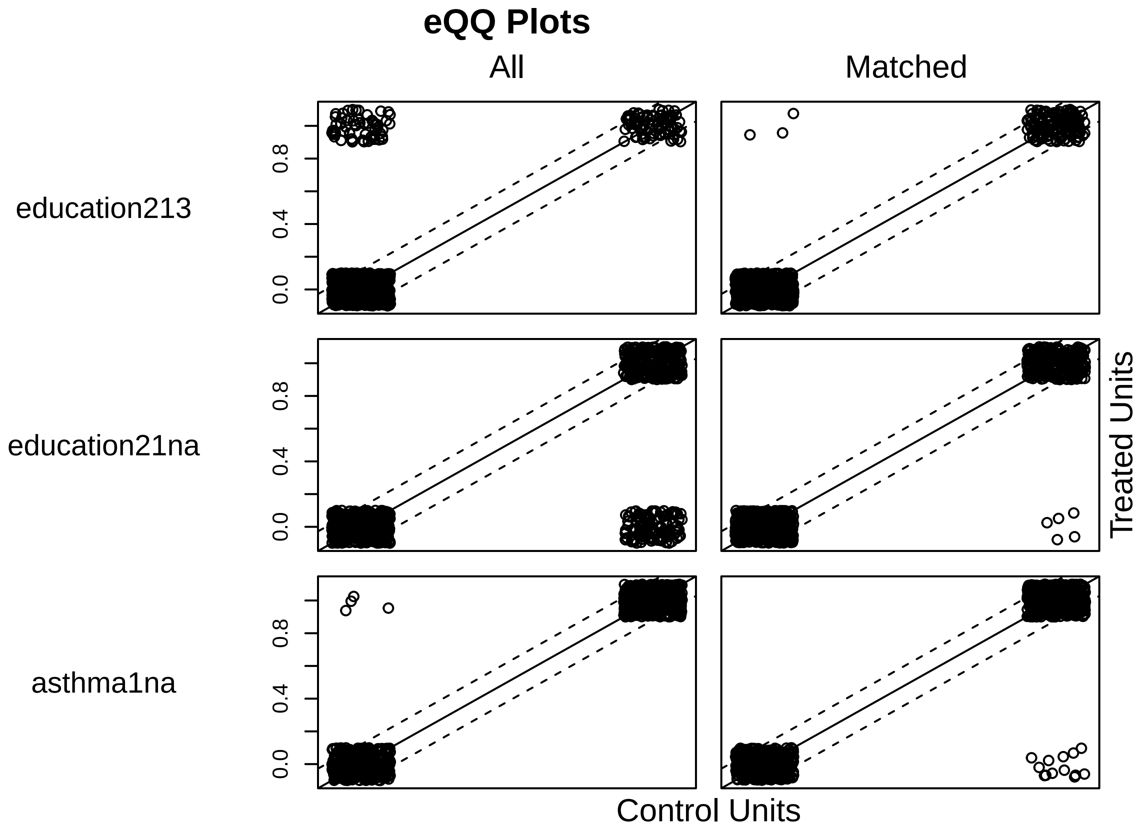
eQQ Plots
All
Matched
education213
education21na
asthma1na
0.0
0.4
0.8
Treated Units
Control Units

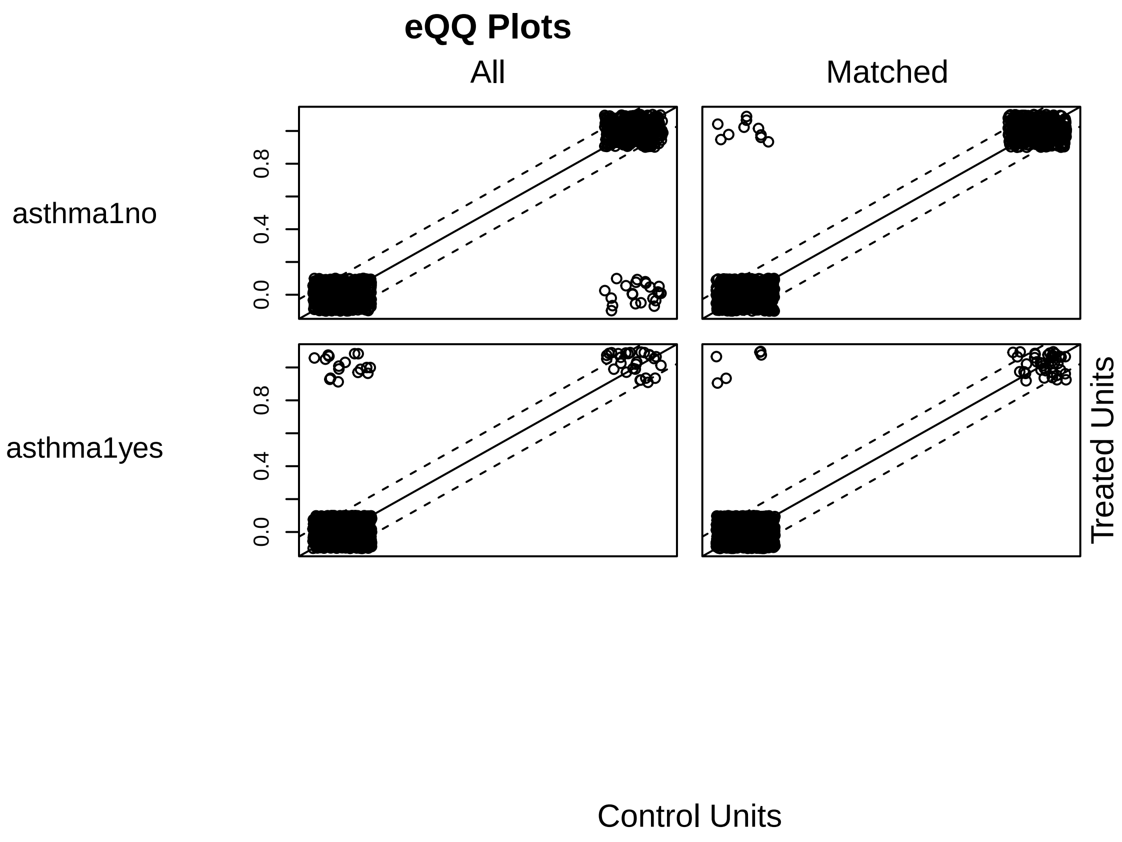


```
plot(summary(psm1))
```

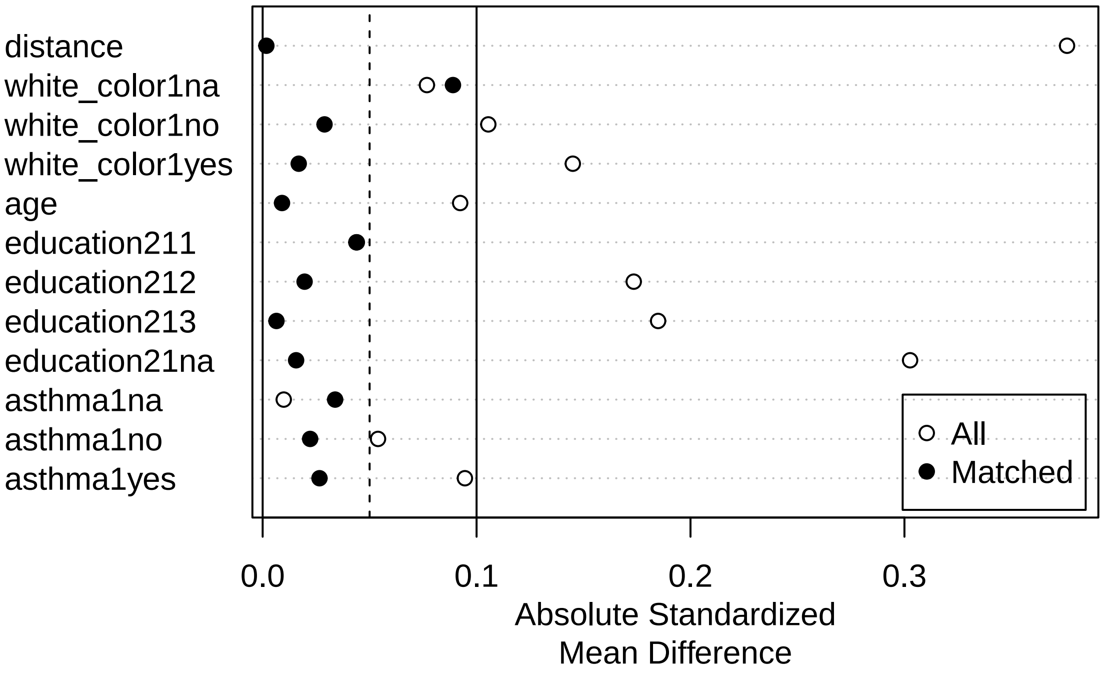


```r
#PSM -  method = "optimal", distance = "logit",
psm2 <- matchit(vaccine1 ~ white_color1 + age + education21 + asthma1, data_final1, method = "optimal",

summary(psm2)
```

```
##
## Call:
## matchit(formula = vaccine1 ~ white_color1 + age + education21 +
##     asthma1, data = data_final1, method = "optimal", distance = "logit",
##     estimand = "ATT")
##
## Summary of Balance for All Data:
##                 Means Treated Means Control Std. Mean Diff. Var. Ratio
## distance               0.2681        0.2399          0.3760     1.1378
## white_color1na         0.0709        0.0906         -0.0768          .
## white_color1no         0.4639        0.5165         -0.1055          .
## white_color1yes        0.4651        0.3928          0.1450          .
## age                   29.8101       30.4358         -0.0923     0.8685
## education211           0.1238        0.1383         -0.0440          .
## education212           0.4026        0.3176          0.1735          .
## education213           0.1671        0.0981          0.1849          .
## education21na          0.3065        0.4460         -0.3026          .
## asthma1na              0.5577        0.5528          0.0099          .
## asthma1no              0.3882        0.4145         -0.0539          .
## asthma1yes             0.0541        0.0327          0.0945          .
##                 eCDF Mean eCDF Max
```

```
## distance           0.1047   0.1801
## white_color1na     0.0197   0.0197
## white_color1no     0.0526   0.0526
## white_color1yes    0.0723   0.0723
## age                0.0144   0.0444
## education211       0.0145   0.0145
## education212       0.0851   0.0851
## education213       0.0690   0.0690
## education21na      0.1395   0.1395
## asthma1na          0.0049   0.0049
## asthma1no          0.0263   0.0263
## asthma1yes         0.0214   0.0214
##
##
## Summary of Balance for Matched Data:
##                 Means Treated Means Control Std. Mean Diff. Var. Ratio
## distance               0.2681        0.2680          0.0008     1.0037
## white_color1na         0.0709        0.0709          0.0000          .
## white_color1no         0.4639        0.5024         -0.0771          .
## white_color1yes        0.4651        0.4267          0.0771          .
## age                   29.8101       29.4772          0.0491     0.9448
## education211           0.1238        0.1130          0.0328          .
## education212           0.4026        0.4062         -0.0074          .
## education213           0.1671        0.1707         -0.0097          .
## education21na          0.3065        0.3101         -0.0078          .
## asthma1na              0.5577        0.5625         -0.0097          .
## asthma1no              0.3882        0.3846          0.0074          .
## asthma1yes             0.0541        0.0529          0.0053          .
##                 eCDF Mean eCDF Max Std. Pair Dist.
## distance           0.0011   0.0060          0.0061
## white_color1na     0.0000   0.0000          0.1250
## white_color1no     0.0385   0.0385          0.7423
## white_color1yes    0.0385   0.0385          0.7711
## age                0.0087   0.0300          0.9008
## education211       0.0108   0.0108          0.5657
## education212       0.0036   0.0036          0.5220
## education213       0.0036   0.0036          0.3899
## education21na      0.0036   0.0036          0.2998
## asthma1na          0.0048   0.0048          0.9486
## asthma1no          0.0036   0.0036          0.8854
## asthma1yes         0.0012   0.0012          0.3135
##
## Percent Balance Improvement:
##                 Std. Mean Diff. Var. Ratio eCDF Mean eCDF Max
## distance                   99.8       97.2      99.0     96.7
## white_color1na            100.0          .     100.0    100.0
## white_color1no             26.9          .      26.9     26.9
## white_color1yes            46.8          .      46.8     46.8
## age                        46.8       59.7      39.8     32.3
## education211               25.4          .      25.4     25.4
## education212               95.8          .      95.8     95.8
## education213               94.8          .      94.8     94.8
## education21na              97.4          .      97.4     97.4
## asthma1na                   1.8          .       1.8      1.8
```

```
## asthma1no                       86.3           .       86.3      86.3
## asthma1yes                      94.4           .       94.4      94.4
##
## Sample Sizes:
##           Control Treated
## All          2538     832
## Matched       832     832
## Unmatched    1706       0
## Discarded       0       0
```

```
plot(psm2, type = "jitter", interactive = FALSE)
```

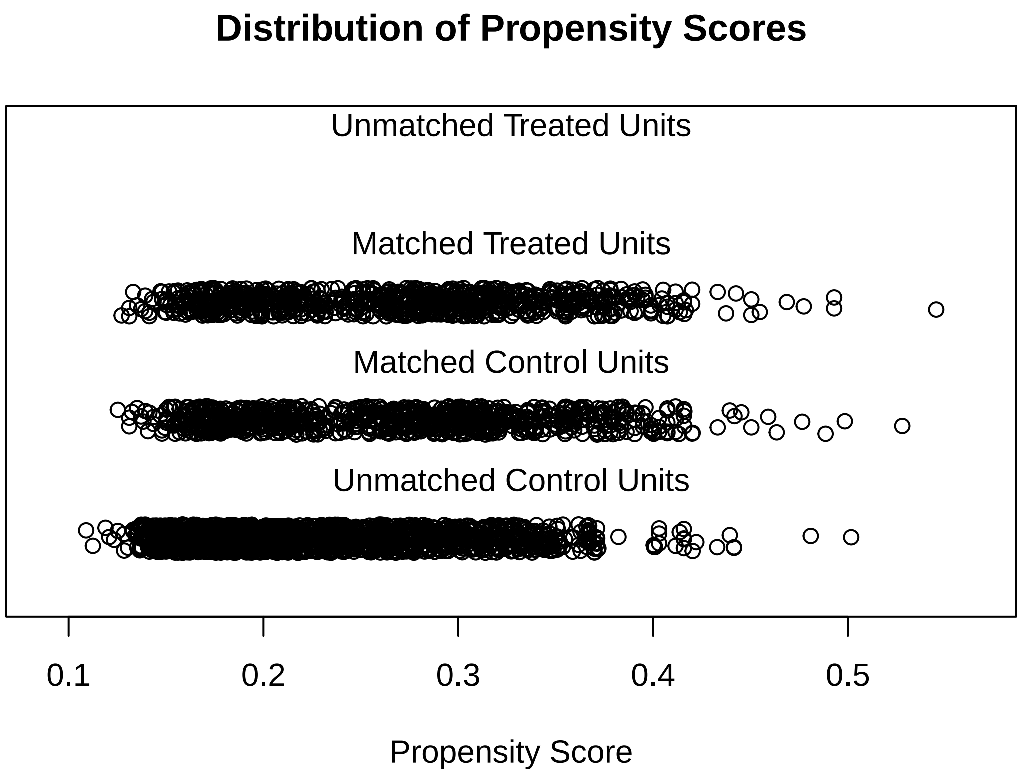


```
plot(psm2, type = "qq", interactive = FALSE,
     which.xs = c("white_color1","age","education21","asthma1"))
```

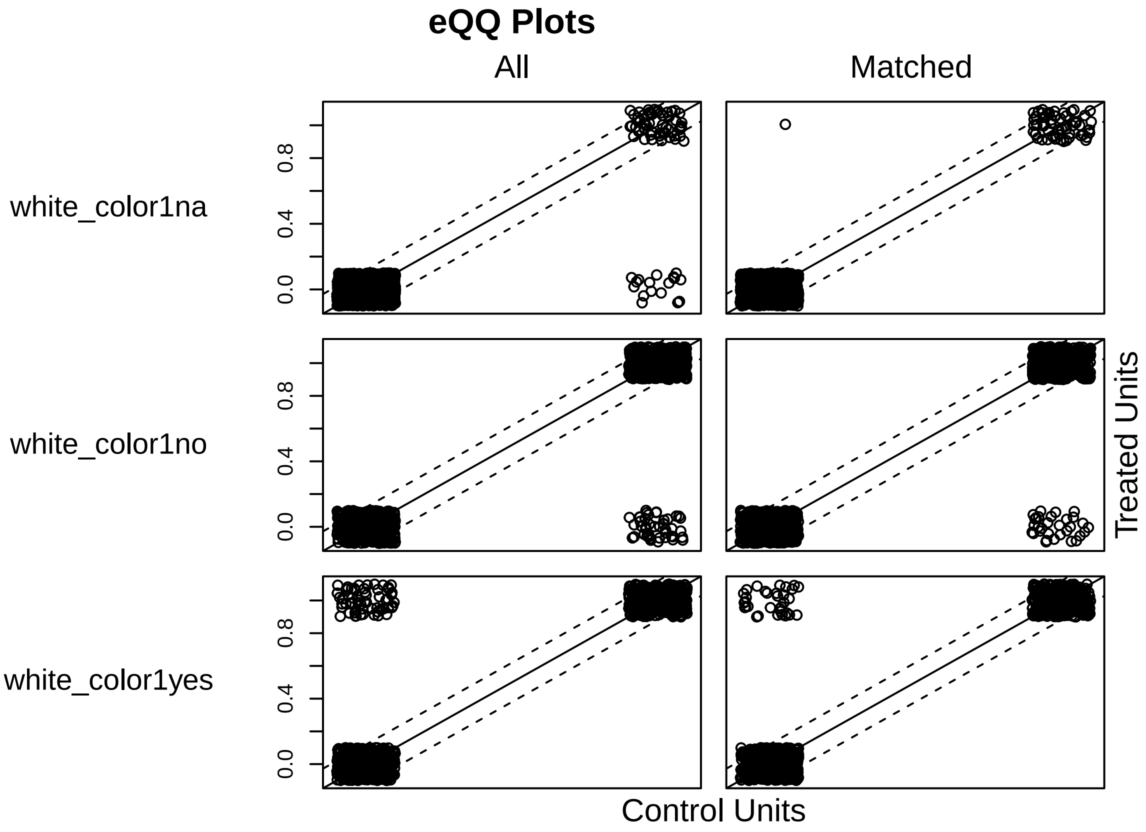
eQQ Plots
All
Matched
white_color1na
white_color1no
white_color1yes
0.0
0.4
0.8
Treated Units
Control Units

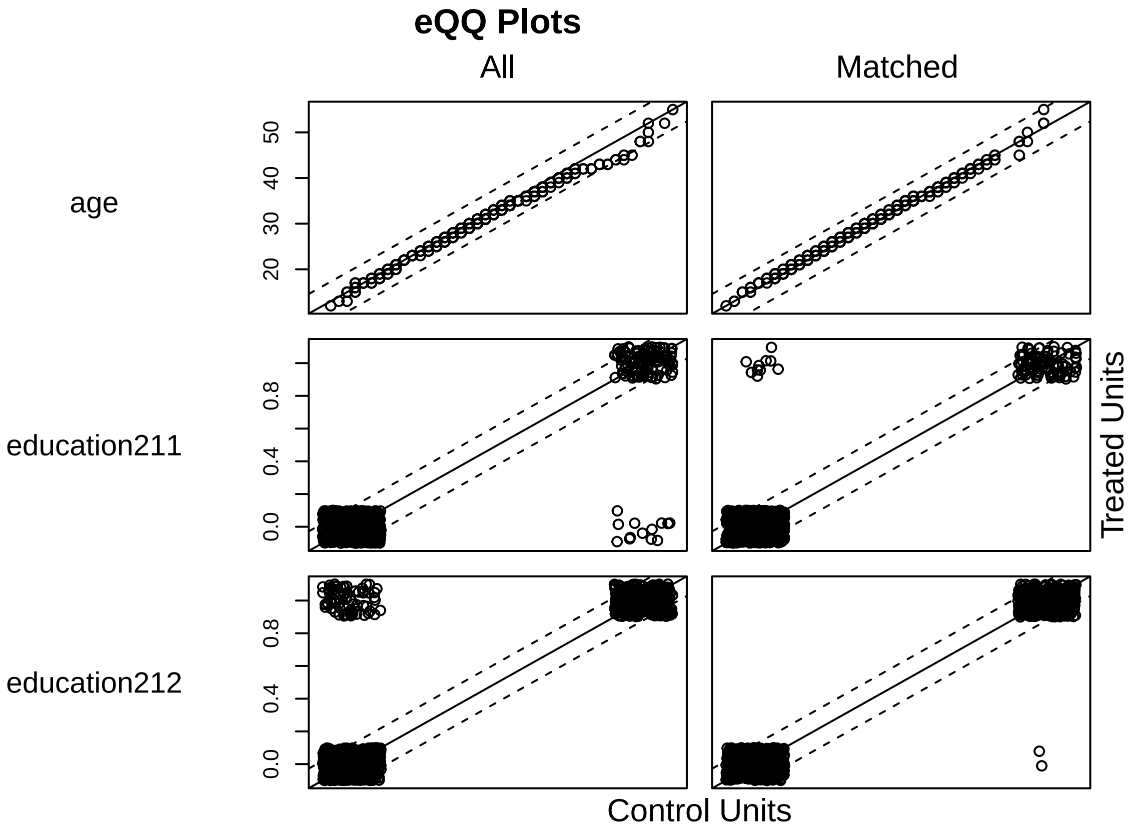
eQQ Plots
All
Matched
age
education211
education212
Treated Units
Control Units
20
30
40
50
0.0
0.4
0.8
0.0
0.4
0.8

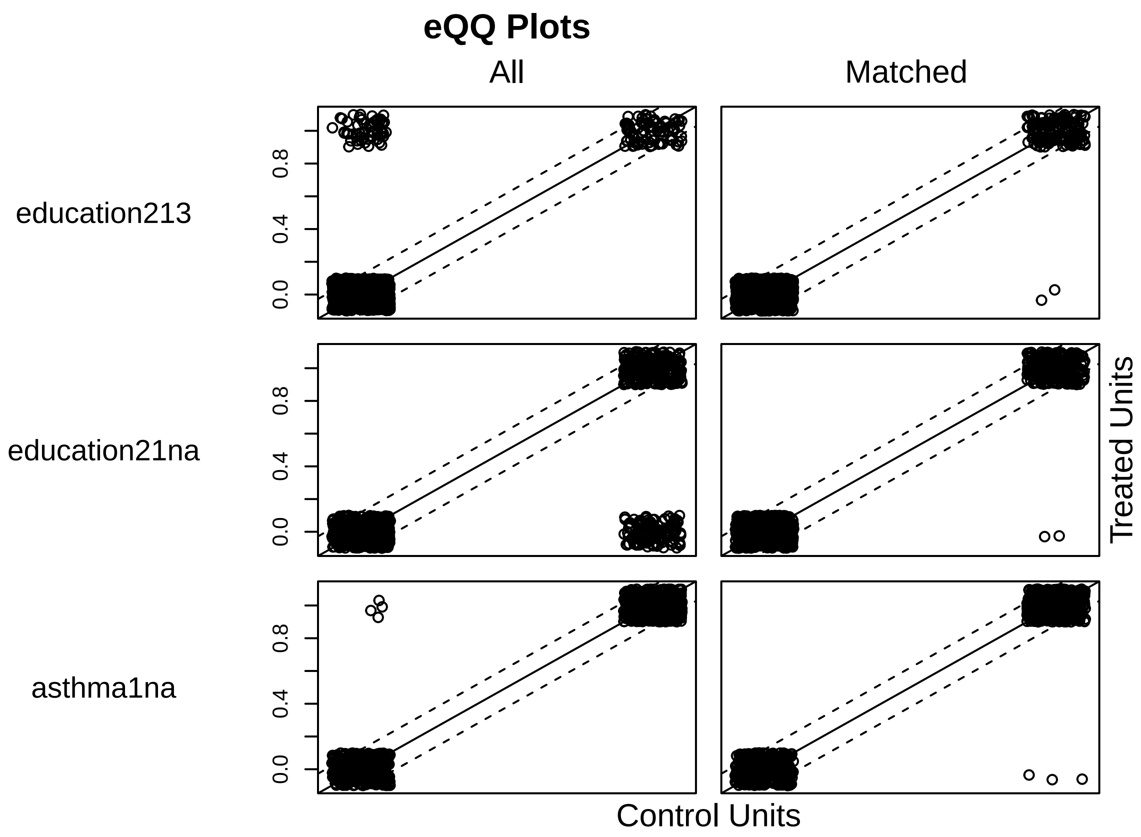
eQQ Plots
All
Matched
education213
education21na
asthma1na
0.0
0.4
0.8
Treated Units
Control Units

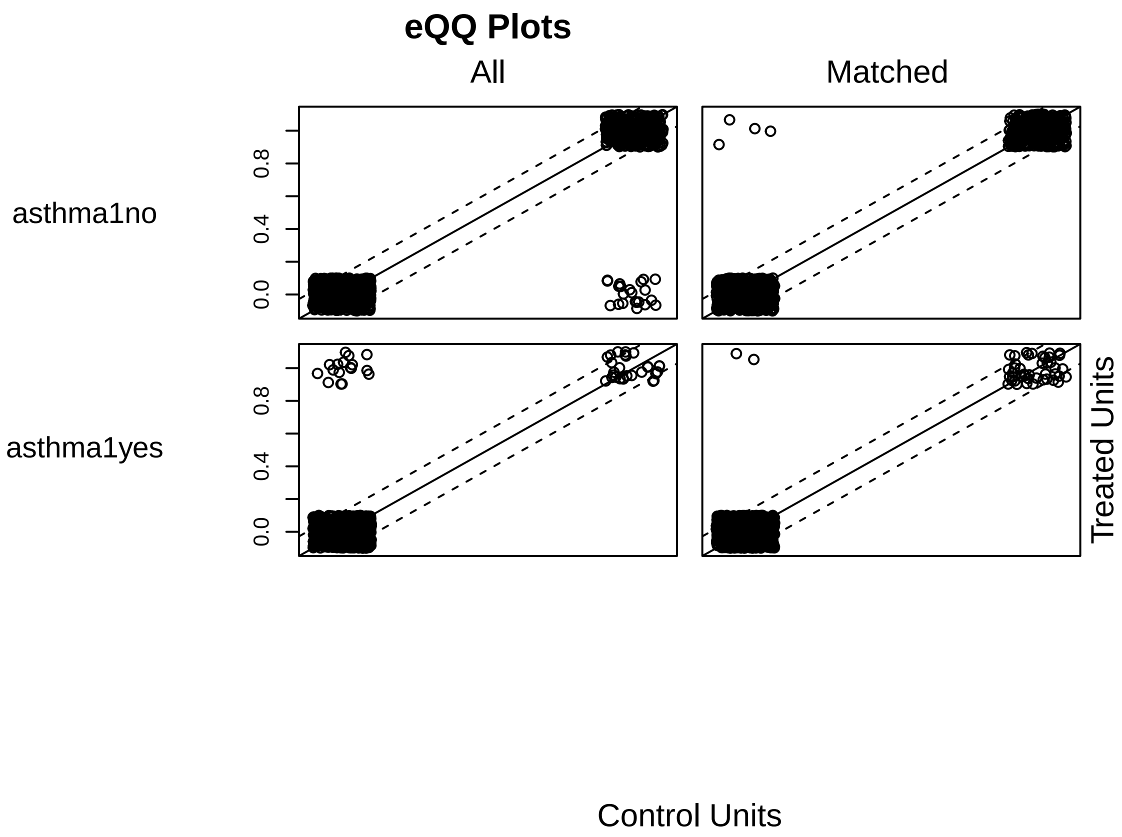


```
plot(summary(psm2))
```

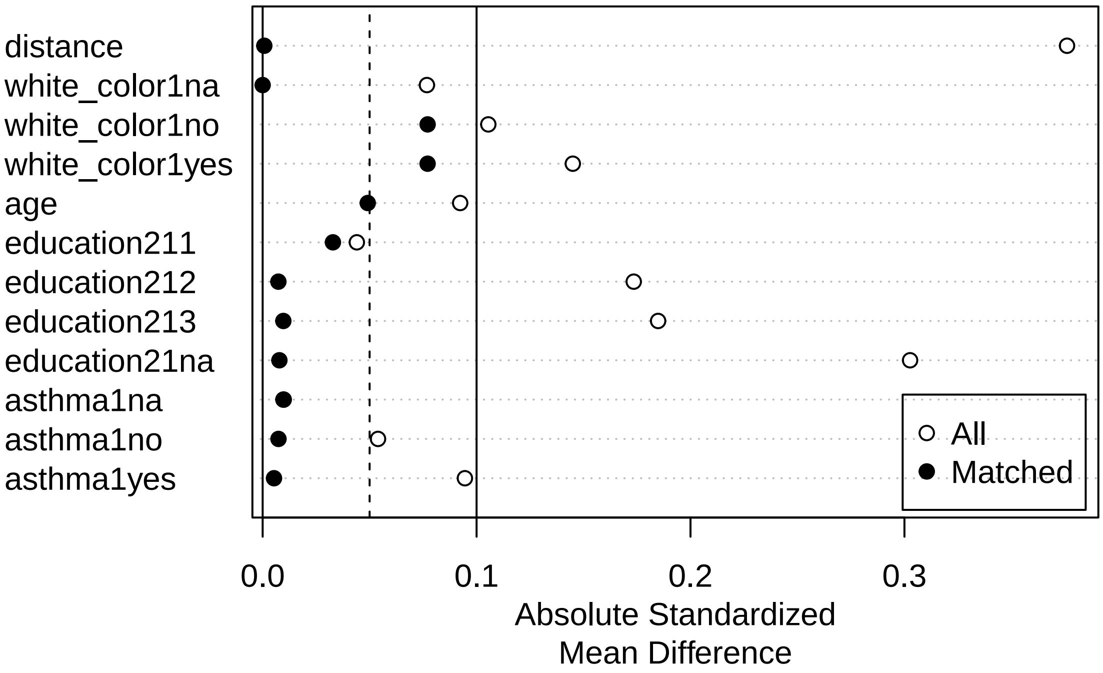

The best is PSM with the nearest method.

```
#Selecting only the selected observations
data_aux <- data_final1[psm1$weights==1, ]

#Now let's join data_aux with data_final

data_psm <- right_join(data_final, data_aux, by= c("id", "vaccine1", "white_color", "age", "education2"

freq(data_psm$vaccine)
```

```
##       n  % val%
## no  832 50   50
## yes 832 50   50
```

For symptoms

Fever

```
with(data_psm, ctable(vaccine, fever, prop = "r", useNA = "no", chisq = TRUE, OR=TRUE))
```

```
## Cross-Tabulation, Row Proportions
## vaccine * fever
```

```
## Data Frame: data_psm
##
##
## --------- ------- ------------- -------------- ---------------
##               fever            no            yes           Total
##   vaccine
##        no           282 (36.0%)    502 (64.0%)    784 (100.0%)
##       yes           255 (33.4%)    509 (66.6%)    764 (100.0%)
##     Total           537 (34.7%)   1011 (65.3%)   1548 (100.0%)
## --------- ------- ------------- -------------- ---------------
##
## ----------------------------
##  Chi.squared   df   p.value
## ------------- ---- ---------
##    1.0362      1    0.3087
## ----------------------------
##
## ----------------------------------
##  Odds Ratio   Lo - 95%   Hi - 95%
## ------------ ---------- ----------
##     1.12        0.91       1.38
## ----------------------------------
```

Cough

```
with(data_psm, ctable(vaccine, cough, prop = "r", useNA = "no", chisq = TRUE, OR=TRUE))
```

```
## Cross-Tabulation, Row Proportions
## vaccine * cough
## Data Frame: data_psm
##
##
## --------- ------- ------------- -------------- ---------------
##               cough            no            yes           Total
##   vaccine
##        no           208 (26.2%)    585 (73.8%)    793 (100.0%)
##       yes           189 (24.2%)    593 (75.8%)    782 (100.0%)
##     Total           397 (25.2%)   1178 (74.8%)   1575 (100.0%)
## --------- ------- ------------- -------------- ---------------
##
## ----------------------------
##  Chi.squared   df   p.value
## ------------- ---- ---------
##    0.7809      1    0.3769
## ----------------------------
##
## ----------------------------------
##  Odds Ratio   Lo - 95%   Hi - 95%
## ------------ ---------- ----------
##     1.12        0.89       1.40
## ----------------------------------
```

Sore throat

```
with(data_psm, ctable(vaccine, sore_throat, prop = "r", useNA = "no", chisq = TRUE, OR=TRUE))
```

```
## Cross-Tabulation, Row Proportions
## vaccine * sore_throat
## Data Frame: data_psm
##
##
## --------- ------------- -------------- ------------- ---------------
##             sore_throat             no           yes           Total
##   vaccine
##        no                  506 (68.6%)   232 (31.4%)    738 (100.0%)
##       yes                  494 (68.0%)   233 (32.0%)    727 (100.0%)
##     Total                 1000 (68.3%)   465 (31.7%)   1465 (100.0%)
## --------- ------------- -------------- ------------- ---------------
##
## ----------------------------
##  Chi.squared   df   p.value
## ------------- ---- ---------
##    0.0384      1    0.8446
## ----------------------------
##
## ----------------------------------
##  Odds Ratio   Lo - 95%   Hi - 95%
## ------------ ---------- ----------
##     1.03        0.83       1.28
## ----------------------------------
```

Dyspnea

```
with(data_psm, ctable(vaccine, dyspnea, prop = "r", useNA = "no", chisq = TRUE, OR=TRUE))
```

```
## Cross-Tabulation, Row Proportions
## vaccine * dyspnea
## Data Frame: data_psm
##
##
## --------- --------- ------------- ------------- ---------------
##             dyspnea            no           yes           Total
##   vaccine
##        no             275 (35.0%)   510 (65.0%)    785 (100.0%)
##       yes             341 (44.9%)   418 (55.1%)    759 (100.0%)
##     Total             616 (39.9%)   928 (60.1%)   1544 (100.0%)
## --------- --------- ------------- ------------- ---------------
##
## ----------------------------
##  Chi.squared   df   p.value
## ------------- ---- ---------
##    15.3488     1     1e-04
```

```
## ----------------------------
##
## ----------------------------------
##  Odds Ratio   Lo - 95%   Hi - 95%
## ------------ ---------- ----------
##     0.66        0.54       0.81
## ----------------------------------
```

Respiratory discomfort

```
with(data_psm, ctable(vaccine, resp_disc, prop = "r", useNA = "no", chisq = TRUE, OR=TRUE))
```

```
## Cross-Tabulation, Row Proportions
## vaccine * resp_disc
## Data Frame: data_psm
##
##
## --------- ----------- ------------- ------------- ---------------
##             resp_disc            no           yes           Total
##   vaccine
##        no               347 (45.5%)   416 (54.5%)    763 (100.0%)
##       yes               337 (45.1%)   411 (54.9%)    748 (100.0%)
##     Total               684 (45.3%)   827 (54.7%)   1511 (100.0%)
## --------- ----------- ------------- ------------- ---------------
##
## ----------------------------
##  Chi.squared   df   p.value
## ------------- ---- ---------
##     0.013      1    0.9091
## ----------------------------
##
## ----------------------------------
##  Odds Ratio   Lo - 95%   Hi - 95%
## ------------ ---------- ----------
##     1.02        0.83       1.25
## ----------------------------------
```

Desaturation

```
with(data_psm, ctable(vaccine, desaturation, prop = "r", useNA = "no", chisq = TRUE, OR=TRUE))
```

```
## Cross-Tabulation, Row Proportions
## vaccine * desaturation
## Data Frame: data_psm
##
##
## --------- -------------- ------------- ------------- ---------------
##             desaturation            no           yes           Total
##   vaccine
```

```
##        no                   408 (53.3%)   358 (46.7%)    766 (100.0%)
##       yes                   491 (66.9%)   243 (33.1%)    734 (100.0%)
##     Total                   899 (59.9%)   601 (40.1%)   1500 (100.0%)
## --------- -------------- ------------- ------------- ---------------
##
## ----------------------------
##  Chi.squared   df   p.value
## ------------- ---- ---------
##    28.4337     1       0
## ----------------------------
##
## ----------------------------------
##  Odds Ratio   Lo - 95%   Hi - 95%
## ------------ ---------- ----------
##     0.56        0.46       0.70
## ----------------------------------
```

Diarrhea

```
with(data_psm, ctable(vaccine, diarrhea, prop = "r", useNA = "no", chisq = TRUE, OR=TRUE))
```

```
## Cross-Tabulation, Row Proportions
## vaccine * diarrhea
## Data Frame: data_psm
##
##
## --------- ---------- -------------- ------------- ---------------
##             diarrhea             no           yes           Total
##   vaccine
##        no                604 (83.3%)   121 (16.7%)    725 (100.0%)
##       yes                595 (83.1%)   121 (16.9%)    716 (100.0%)
##     Total               1199 (83.2%)   242 (16.8%)   1441 (100.0%)
## --------- ---------- -------------- ------------- ---------------
##
## ----------------------------
##  Chi.squared   df   p.value
## ------------- ---- ---------
##    0.0013      1    0.9712
## ----------------------------
##
## ----------------------------------
##  Odds Ratio   Lo - 95%   Hi - 95%
## ------------ ---------- ----------
##     1.02        0.77       1.34
## ----------------------------------
```

Vomit

```
with(data_psm, ctable(vaccine, vomit, prop = "r", useNA = "no", chisq = TRUE, OR=TRUE))
```

```
## Cross-Tabulation, Row Proportions
## vaccine * vomit
## Data Frame: data_psm
##
##
## --------- ------- -------------- ------------- ---------------
##             vomit             no           yes           Total
##   vaccine
##        no            618 (85.6%)   104 (14.4%)    722 (100.0%)
##       yes            609 (84.8%)   109 (15.2%)    718 (100.0%)
##     Total           1227 (85.2%)   213 (14.8%)   1440 (100.0%)
## --------- ------- -------------- ------------- ---------------
##
## ----------------------------
##  Chi.squared   df   p.value
## ------------- ---- ---------
##    0.1162      1    0.7332
## ----------------------------
##
## ----------------------------------
##  Odds Ratio   Lo - 95%   Hi - 95%
## ------------ ---------- ----------
##     1.06        0.80       1.42
## ----------------------------------
```

Abdominal pain

```
with(data_psm, ctable(vaccine, abd_pain, prop = "r", useNA = "no", chisq = TRUE, OR=TRUE))
```

```
## Cross-Tabulation, Row Proportions
## vaccine * abd_pain
## Data Frame: data_psm
##
##
## --------- ---------- ------------- ------------- ---------------
##             abd_pain            no           yes           Total
##   vaccine
##        no               564 (87.7%)    79 (12.3%)    643 (100.0%)
##       yes               384 (87.3%)    56 (12.7%)    440 (100.0%)
##     Total               948 (87.5%)   135 (12.5%)   1083 (100.0%)
## --------- ---------- ------------- ------------- ---------------
##
## ----------------------------
##  Chi.squared   df   p.value
## ------------- ---- ---------
##    0.0149      1    0.9028
## ----------------------------
##
## ----------------------------------
##  Odds Ratio   Lo - 95%   Hi - 95%
## ------------ ---------- ----------
##     1.04        0.72       1.50
```

```
## ----------------------------------
```

Fatigue

```r
with(data_psm, ctable(vaccine, fatigue, prop = "r", useNA = "no", chisq = TRUE, OR=TRUE))
```

```
## Cross-Tabulation, Row Proportions
## vaccine * fatigue
## Data Frame: data_psm
##
##
## --------- --------- ------------- ------------- ---------------
##             fatigue            no           yes           Total
##   vaccine
##        no             428 (65.0%)   230 (35.0%)    658 (100.0%)
##       yes             290 (64.4%)   160 (35.6%)    450 (100.0%)
##     Total             718 (64.8%)   390 (35.2%)   1108 (100.0%)
## --------- --------- ------------- ------------- ---------------
##
## ----------------------------
##  Chi.squared   df   p.value
## ------------- ---- ---------
##    0.0201      1    0.8873
## ----------------------------
##
## ----------------------------------
##  Odds Ratio   Lo - 95%   Hi - 95%
## ------------ ---------- ----------
##     1.03        0.80       1.32
## ----------------------------------
```

Olfactory loss

```r
with(data_psm, ctable(vaccine, olfac_loss, prop = "r", useNA = "no", chisq = TRUE, OR=TRUE))
```

```
## Cross-Tabulation, Row Proportions
## vaccine * olfac_loss
## Data Frame: data_psm
##
##
## --------- ------------ ------------- ------------- ---------------
##             olfac_loss            no           yes           Total
##   vaccine
##        no                511 (78.4%)   141 (21.6%)    652 (100.0%)
##       yes                320 (68.8%)   145 (31.2%)    465 (100.0%)
##     Total                831 (74.4%)   286 (25.6%)   1117 (100.0%)
## --------- ------------ ------------- ------------- ---------------
##
## ----------------------------
```

```
##   Chi.squared   df   p.value
## ------------- ---- ---------
##    12.5178     1     4e-04
## ----------------------------
##
## ----------------------------------
##   Odds Ratio   Lo - 95%   Hi - 95%
## ------------ ---------- ----------
##     1.64        1.25       2.15
## ----------------------------------
```

Loss of taste

```
with(data_psm, ctable(vaccine, loss_taste, prop = "r", useNA = "no", chisq = TRUE, OR=TRUE))
```

```
## Cross-Tabulation, Row Proportions
## vaccine * loss_taste
## Data Frame: data_psm
##
##
## --------- ------------ ------------- ------------- ---------------
##             loss_taste            no           yes           Total
##   vaccine
##        no                519 (79.2%)   136 (20.8%)    655 (100.0%)
##       yes                324 (70.3%)   137 (29.7%)    461 (100.0%)
##     Total                843 (75.5%)   273 (24.5%)   1116 (100.0%)
## --------- ------------ ------------- ------------- ---------------
##
## ----------------------------
##   Chi.squared   df   p.value
## ------------- ---- ---------
##    11.2616     1     8e-04
## ----------------------------
##
## ----------------------------------
##   Odds Ratio   Lo - 95%   Hi - 95%
## ------------ ---------- ----------
##     1.61        1.23       2.12
## ----------------------------------
```

Any respiratory symptom

```
with(data_psm, ctable(vaccine, resp_symp, prop = "r", useNA = "no", chisq = TRUE, OR=TRUE))
```

```
## Cross-Tabulation, Row Proportions
## vaccine * resp_symp
## Data Frame: data_psm
##
##
```

```
## --------- ----------- ------------- -------------- ---------------
##              resp_symp            no            yes           Total
##   vaccine
##        no                167 (20.7%)    641 (79.3%)    808 (100.0%)
##       yes                213 (27.2%)    570 (72.8%)    783 (100.0%)
##     Total                380 (23.9%)   1211 (76.1%)   1591 (100.0%)
## --------- ----------- ------------- -------------- ---------------
##
## ----------------------------
##  Chi.squared   df   p.value
## ------------- ---- ---------
##    8.9846      1    0.0027
## ----------------------------
##
## ----------------------------------
##  Odds Ratio   Lo - 95%   Hi - 95%
## ------------ ---------- ----------
##     0.70        0.55       0.88
## ----------------------------------
```

Any symptom

```
with(data_psm, ctable(vaccine, symptom, prop = "r", useNA = "no", chisq = TRUE, OR=TRUE))
```

```
## Cross-Tabulation, Row Proportions
## vaccine * symptom
## Data Frame: data_psm
##
##
## --------- --------- ----------- -------------- ---------------
##              symptom          no            yes           Total
##   vaccine
##        no              31 (3.8%)    794 (96.2%)    825 (100.0%)
##       yes              35 (4.3%)    781 (95.7%)    816 (100.0%)
##     Total              66 (4.0%)   1575 (96.0%)   1641 (100.0%)
## --------- --------- ----------- -------------- ---------------
##
## ----------------------------
##  Chi.squared   df   p.value
## ------------- ---- ---------
##    0.1784      1    0.6727
## ----------------------------
##
## ----------------------------------
##  Odds Ratio   Lo - 95%   Hi - 95%
## ------------ ---------- ----------
##     0.87        0.53       1.43
## ----------------------------------
```

For outcomes

Hospitalization

```r
with(data_psm, ctable(vaccine, hospitalization, prop = "r", useNA = "no", chisq = TRUE, OR=TRUE))
```

```
## Cross-Tabulation, Row Proportions
## vaccine * hospitalization
## Data Frame: data_psm
##
##
## --------- ----------------- ----------- -------------- ---------------
##           hospitalization          no            yes           Total
##   vaccine
##        no                  21 (2.5%)    806 (97.5%)    827 (100.0%)
##       yes                  35 (4.2%)    789 (95.8%)    824 (100.0%)
##     Total                  56 (3.4%)   1595 (96.6%)   1651 (100.0%)
## --------- ----------------- ----------- -------------- ---------------
##
## ----------------------------
##  Chi.squared   df   p.value
## ------------- ---- ---------
##    3.1729      1    0.0749
## ----------------------------
##
## ----------------------------------
##  Odds Ratio   Lo - 95%   Hi - 95%
## ------------ ---------- ----------
##     0.59        0.34       1.02
## ----------------------------------
```

ICU

```r
with(data_psm, ctable(vaccine, icu, prop = "r", useNA = "no", chisq = TRUE, OR=TRUE))
```

```
## Cross-Tabulation, Row Proportions
## vaccine * icu
## Data Frame: data_psm
##
##
## --------- ----- -------------- ------------- ---------------
##           icu             no           yes           Total
##   vaccine
##        no          545 (69.7%)   237 (30.3%)    782 (100.0%)
##       yes          597 (78.9%)   160 (21.1%)    757 (100.0%)
##     Total         1142 (74.2%)   397 (25.8%)   1539 (100.0%)
## --------- ----- -------------- ------------- ---------------
##
## ----------------------------
##  Chi.squared   df   p.value
```

```
## ------------- ---- ---------
##    16.4249     1     1e-04
## ----------------------------
##
## ----------------------------------
##  Odds Ratio   Lo - 95%   Hi - 95%
## ------------ ---------- ----------
##     0.62        0.49       0.78
## ----------------------------------
```

Length time in ICU

```
data_psm_aux <- data_psm %>%
  filter (icu == "yes")
```

```
datasummary((vaccine) ~ time_icu*(n+media+DP+mediana+q25+q75+IQR),
            data = data_psm_aux, output = 'markdown')
```

	n	media	DP	mediana	q25	q75	IQR
no	152.00	11.93	13.40	7.00	3.00	15.00	12.00
yes	110.00	11.83	11.25	8.00	4.00	17.00	13.00

```
#t-test
t.test(time_icu ~ vaccine, data = data_psm_aux)
```

```
##
##  Welch Two Sample t-test
##
## data:  time_icu by vaccine
## t = 0.065737, df = 254.27, p-value = 0.9476
## alternative hypothesis: true difference in means between group no and group yes is not equal to 0
## 95 percent confidence interval:
##  -2.906197  3.106914
## sample estimates:
##  mean in group no mean in group yes
##          11.92763          11.82727
```

Ventilatory support

```
with(data_psm, ctable(vaccine, ventilatory_support, prop = "r", useNA = "no", chisq = TRUE, OR=TRUE))
```

```
## Cross-Tabulation, Row Proportions
## vaccine * ventilatory_support
## Data Frame: data_psm
##
##
## --------- --------------------- ------------- ------------- -------------- ---------------
```

```
##              ventilatory_support      invasive            no   non-invasive           Total
##   vaccine
##        no                         122 (15.5%)   353 (44.9%)    311 (39.6%)    786 (100.0%)
##       yes                          68 ( 8.8%)   450 (58.5%)    251 (32.6%)    769 (100.0%)
##     Total                         190 (12.2%)   803 (51.6%)    562 (36.1%)   1555 (100.0%)
## --------- --------------------- ------------- ------------- -------------- ---------------
##
## ----------------------------
##  Chi.squared   df   p.value
## ------------- ---- ---------
##    33.2885     2       0
## ----------------------------
```

Intubation

```
with(data_psm, ctable(vaccine, intubation, prop = "r", useNA = "no", chisq = TRUE, OR=TRUE))
```

```
## Cross-Tabulation, Row Proportions
## vaccine * intubation
## Data Frame: data_psm
##
##
## --------- ------------ -------------- ------------- ---------------
##              intubation             no           yes           Total
##   vaccine
##        no                664 (84.5%)   122 (15.5%)    786 (100.0%)
##       yes                701 (91.2%)    68 ( 8.8%)    769 (100.0%)
##     Total               1365 (87.8%)   190 (12.2%)   1555 (100.0%)
## --------- ------------ -------------- ------------- ---------------
##
## ----------------------------
##  Chi.squared   df   p.value
## ------------- ---- ---------
##    15.5497     1     1e-04
## ----------------------------
##
## ----------------------------------
##  Odds Ratio   Lo - 95%   Hi - 95%
## ------------ ---------- ----------
##     0.53        0.39       0.72
## ----------------------------------
```

Death

```
with(data_psm, ctable(vaccine, death, prop = "r", useNA = "no", chisq = TRUE, OR=TRUE))
```

```
## Cross-Tabulation, Row Proportions
## vaccine * death
## Data Frame: data_psm
```

```
##
##
## --------- ------- -------------- ------------- ---------------
##             death           cure         death           Total
##   vaccine
##        no              711 (85.5%)   121 (14.5%)    832 (100.0%)
##       yes              788 (94.7%)    44 ( 5.3%)    832 (100.0%)
##     Total             1499 (90.1%)   165 ( 9.9%)   1664 (100.0%)
## --------- ------- -------------- ------------- ---------------
##
## ----------------------------
##  Chi.squared   df    p.value
## ------------- ---- ---------
##    38.8593     1        0
## ----------------------------
##
## ----------------------------------
##  Odds Ratio   Lo - 95%   Hi - 95%
## ------------ ---------- ----------
##     0.33         0.23       0.47
## ----------------------------------
```

Vaccination and mortality maps

```
data_final_vac <- data_final %>%
 filter(vaccine == "no")

valor_vac <- data.frame(table(data_final_vac$SG_UF))
colnames(valor_vac) <- c("uf", "n_vac")

valor <- data.frame(table(data_final$SG_UF))
colnames(valor) <- c("uf", "n")

dt_estado <- left_join(valor, valor_vac, by= "uf")

dt_estado  <- dt_estado %>%
  mutate(n_vac = ifelse(is.na(n_vac), 0, n_vac))

dt_estado  <- dt_estado %>%
  mutate(T1 = (n_vac/n)*100)

dt_estado1 <- dt_estado
dt_estado1 <- dt_estado1 %>%
  rename(T2=T1)

print(dt_estado)
```

```
##    uf    n n_vac        T1
## 1  AC    5     4  80.00000
## 2  AL    8     8 100.00000
## 3  AM  143   122  85.31469
```

```
## 4  AP   14    8  57.14286
## 5  BA   57   51  89.47368
## 6  CE  108   88  81.48148
## 7  DF   32   28  87.50000
## 8  ES   20   19  95.00000
## 9  GO  111   81  72.97297
## 10 MA   50   45  90.00000
## 11 MG  293  231  78.83959
## 12 MS  104   90  86.53846
## 13 MT  131  100  76.33588
## 14 PA  162  130  80.24691
## 15 PB   89   81  91.01124
## 16 PE  134   92  68.65672
## 17 PI   48   26  54.16667
## 18 PR  229  175  76.41921
## 19 RJ  124  102  82.25806
## 20 RN   44   27  61.36364
## 21 RO   72   56  77.77778
## 22 RR    3    3 100.00000
## 23 RS  201  125  62.18905
## 24 SC  110   79  71.81818
## 25 SE   10    7  70.00000
## 26 SP 1066  758  71.10694
## 27 TO    2    2 100.00000
```

```
dt <- rbind(c("AC",12), c("AL",27), c("AP",16), c("AM",13), c("BA",29),
              c("CE",23), c("DF",53), c("ES",32), c("GO",52), c("MA",21),
              c("MT",51), c("MS",50), c("MG",31), c("PA",15), c("PB",25),
              c("PR",41), c("PE",26), c("PI",22), c("RN",24), c("RS",43),
              c("RJ",33), c("RO",11), c("RR",14), c("SC",42), c("SP",35),
              c("SE",28), c("TO",17)) %>% data.table %>% `colnames<-`(c("uf","id"))

mapaUF <- readRDS("mapaUF.Rds")

dt2 <- full_join(dt, dt_estado, by = "uf")
g1 <- ggplot(dt2) +
  geom_map(map = mapaUF, color = 'gray30', aes_string(map_id = "id", fill = "T1")) +
  geom_path(data = mapaUF, color = 'gray30', size = .1, aes(x = long, y = lat, group = group)) +
  theme_void() + coord_equal() +
  labs(fill = "Not-vaccinated rate", title = "")  +
  scale_fill_distiller(palette="Purples", trans="reverse", limits = c(100,0))

g1
```

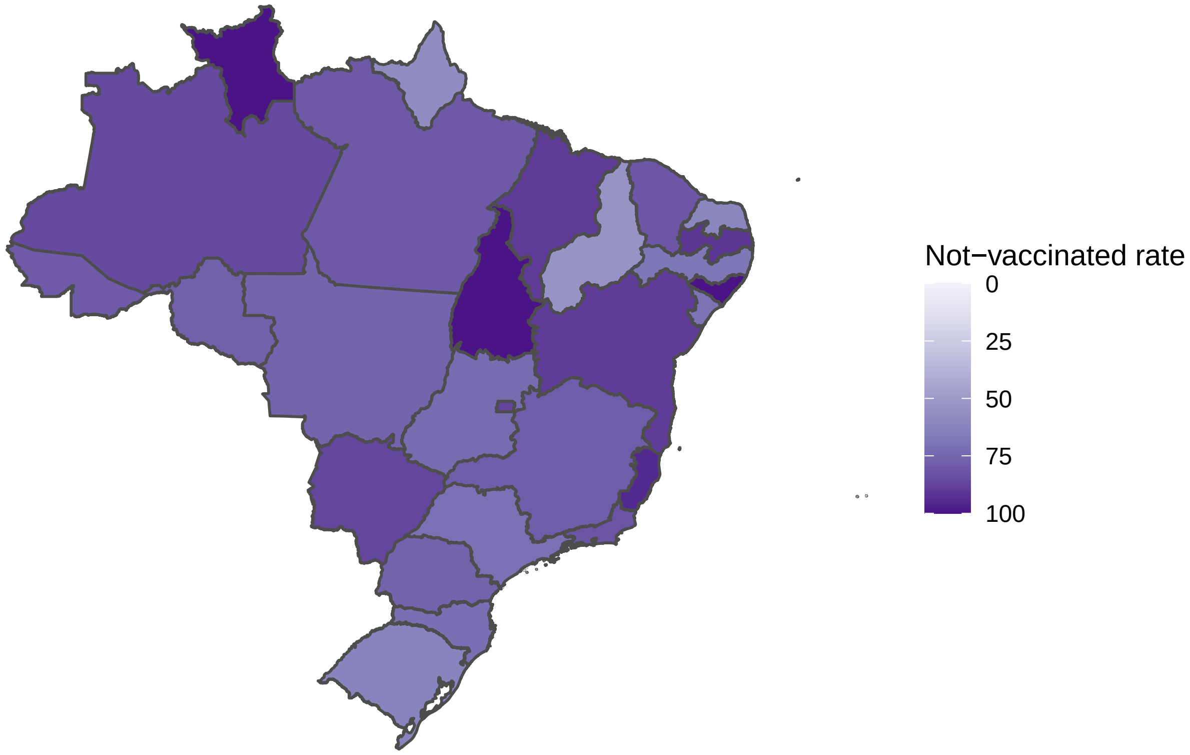


```
data_final_death <- data_final %>%
 filter(death == "death")

valor_death <- data.frame(table(data_final_death$SG_UF))
colnames(valor_death) <- c("uf", "n_death")

valor <- data.frame(table(data_final$SG_UF))
colnames(valor) <- c("uf", "n")

dt_estado <- left_join(valor, valor_death, by= "uf")

dt_estado  <- dt_estado %>%
  mutate(n_death = ifelse(is.na(n_death), 0, n_death))

dt_estado  <- dt_estado %>%
  mutate(T1 = (n_death/n)*100)

print(dt_estado)
```

```
##    uf   n n_death        T1
## 1  AC   5       2 40.000000
## 2  AL   8       2 25.000000
## 3  AM 143      22 15.384615
## 4  AP  14       0  0.000000
## 5  BA  57       2  3.508772
```

```
## 6  CE  108       10  9.259259
## 7  DF   32        4 12.500000
## 8  ES   20        7 35.000000
## 9  GO  111       24 21.621622
## 10 MA   50       17 34.000000
## 11 MG  293       31 10.580205
## 12 MS  104       12 11.538462
## 13 MT  131        9  6.870229
## 14 PA  162       20 12.345679
## 15 PB   89       13 14.606742
## 16 PE  134        6  4.477612
## 17 PI   48        3  6.250000
## 18 PR  229       23 10.043668
## 19 RJ  124       22 17.741935
## 20 RN   44        8 18.181818
## 21 RO   72       11 15.277778
## 22 RR    3        2 66.666667
## 23 RS  201       19  9.452736
## 24 SC  110       10  9.090909
## 25 SE   10        0  0.000000
## 26 SP 1066       89  8.348968
## 27 TO    2        1 50.000000
```

```
dt <- rbind(c("AC",12), c("AL",27), c("AP",16), c("AM",13), c("BA",29),
              c("CE",23), c("DF",53), c("ES",32), c("GO",52), c("MA",21),
              c("MT",51), c("MS",50), c("MG",31), c("PA",15), c("PB",25),
              c("PR",41), c("PE",26), c("PI",22), c("RN",24), c("RS",43),
              c("RJ",33), c("RO",11), c("RR",14), c("SC",42), c("SP",35),
              c("SE",28), c("TO",17)) %>% data.table %>% `colnames<-`(c("uf","id"))

mapaUF <- readRDS("mapaUF.Rds")

dt2 <- full_join(dt, dt_estado, by = "uf")
g2 <- ggplot(dt2) +
  geom_map(map = mapaUF, color = 'gray30', aes_string(map_id = "id", fill = "T1")) +
  geom_path(data = mapaUF, color = 'gray30', size = .1, aes(x = long, y = lat, group = group)) +
  theme_void() + coord_equal() +
  labs(fill = "Death rate", title = "")  +
  scale_fill_distiller(palette="Purples", trans="reverse", limits = c(100,0))

g2
```

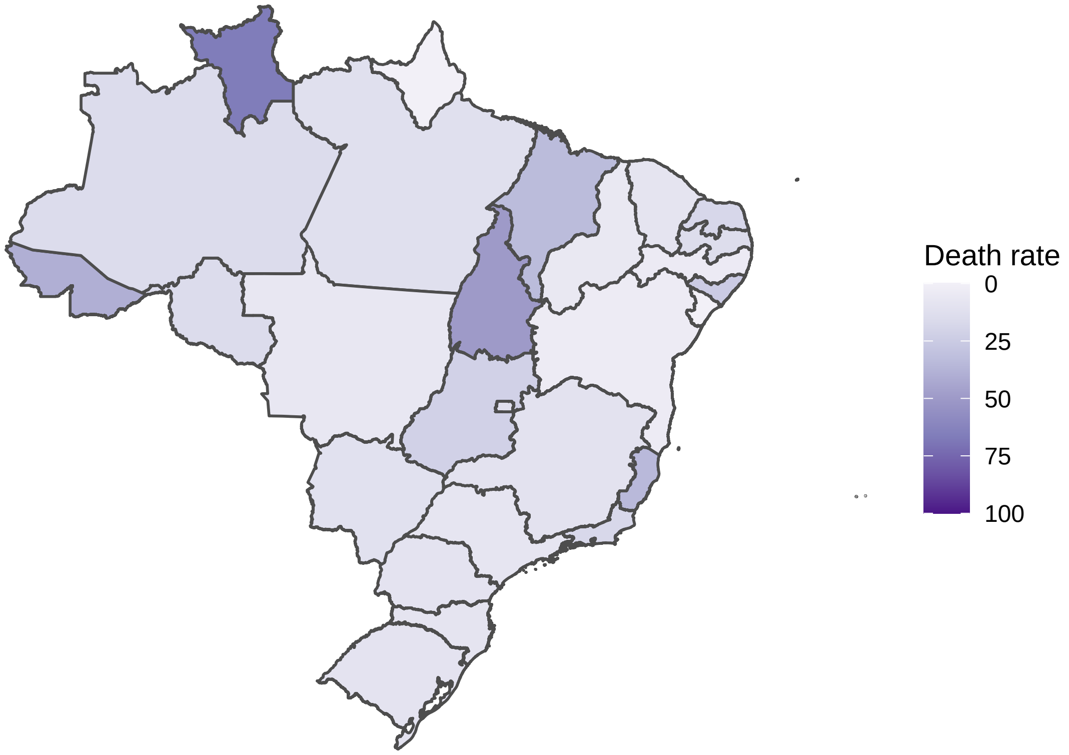

Spearman correlation between % unvaccinated and % death:

```
dt_cor <- full_join(dt_estado, dt_estado1, by = "uf")

SpearmanRho(dt_cor$T1, dt_cor$T2, conf.level=0.95)
```

```
##       rho    lwr.ci    upr.ci
## 0.6409043 0.3448889 0.8209691
```

```
cor.test(dt_cor$T1, dt_cor$T2, method = "spearman")
```

```
##
##  Spearman's rank correlation rho
##
## data:  dt_cor$T1 and dt_cor$T2
## S = 1176.4, p-value = 0.000316
## alternative hypothesis: true rho is not equal to 0
## sample estimates:
##       rho
## 0.6409043
```
